# The Intrinsically Disordered Regions of the *Drosophila melanogaster* Hox Protein Ultrabithorax Select Interacting Proteins Based on Partner Topology

**DOI:** 10.1371/journal.pone.0108217

**Published:** 2014-10-06

**Authors:** Hao-Ching Hsiao, Kim L. Gonzalez, Daniel J. Catanese, Kristopher E. Jordy, Kathleen S. Matthews, Sarah E. Bondos

**Affiliations:** 1 Reynolds Medical Building, Department of Molecular and Cellular Medicine, Texas A&M Health Science Center, College Station, Texas, United States of America; 2 Department of Biochemistry and Cell Biology, Rice University, Houston, Texas, United States of America; Weizmann Institute of Science, Israel

## Abstract

Interactions between structured proteins require a complementary topology and surface chemistry to form sufficient contacts for stable binding. However, approximately one third of protein interactions are estimated to involve intrinsically disordered regions of proteins. The dynamic nature of disordered regions before and, in some cases, after binding calls into question the role of partner topology in forming protein interactions. To understand how intrinsically disordered proteins identify the correct interacting partner proteins, we evaluated interactions formed by the *Drosophila melanogaster* Hox transcription factor Ultrabithorax (Ubx), which contains both structured and disordered regions. Ubx binding proteins are enriched in specific folds: 23 of its 39 partners include one of 7 folds, out of the 1195 folds recognized by SCOP. For the proteins harboring the two most populated folds, DNA-RNA binding 3-helical bundles and α-α superhelices, the regions of the partner proteins that exhibit these preferred folds are sufficient for Ubx binding. Three disorder-containing regions in Ubx are required to bind these partners. These regions are either alternatively spliced or multiply phosphorylated, providing a mechanism for cellular processes to regulate Ubx-partner interactions. Indeed, partner topology correlates with the ability of individual partner proteins to bind Ubx spliceoforms. Partners bind different disordered regions within Ubx to varying extents, creating the potential for competition between partners and cooperative binding by partners. The ability of partners to bind regions of Ubx that activate transcription and regulate DNA binding provides a mechanism for partners to modulate transcription regulation by Ubx, and suggests that one role of disorder in Ubx is to coordinate multiple molecular functions in response to tissue-specific cues.

## Introduction

Most biological processes are implemented and regulated by macromolecular complexes, in which proteins are major components. The function of an individual protein, therefore, is often determined by the identity and range of the proteins to which it binds [1]–[3]. Consequently, proteins must specifically and reliably bind the correct partners *in vivo* [4]–[7]. Interactions between structured proteins require complementary topologies that generate sufficient interfacial surface area [8]–[11] and complementary surface chemical groups capable of creating stable interprotein bonds [11]–[13]. Residues forming an interface between two structured proteins are often less dynamic relative to non-interfacial surface residues, even when the proteins are in the unbound state [8].

Intrinsically disordered proteins and protein regions are present in more than one third of protein complexes and are enriched in proteins with multiple partners [14]–[24]. As monomers, these proteins lack stable globular structures and rapidly interconvert among a large ensemble of conformations. Disordered protein monomers can sample structure present in the bound complex or be extremely dynamic with little detectable canonical structure [25]–[27]. The disordered region may fold to similar structures present in all interactions, or a single disordered region may adopt many different structures to bind protein partners with different topologies [28]–[33].

In contrast to the complementary interface formed by two folded proteins, a subset of disordered regions remain highly dynamic even when bound, either initially through an induced-fit binding mechanism [30], [34] or as part of a heterogeneous final complex [35]–[39]. This structural heterogeneity in the complex has been proposed to be an essential component of fine-tuning the function of the complex [35] as well as maintaining the sensitivity of the complex to evolving cellular signals [40]. The extreme malleability of intrinsically disordered regions, even in the bound state, raises questions regarding the role of the structure and surface topology of the partner protein in these interactions. Indeed, disordered proteins bind more types of protein structures (folds) than do structured proteins [41].

**Figure 1:**
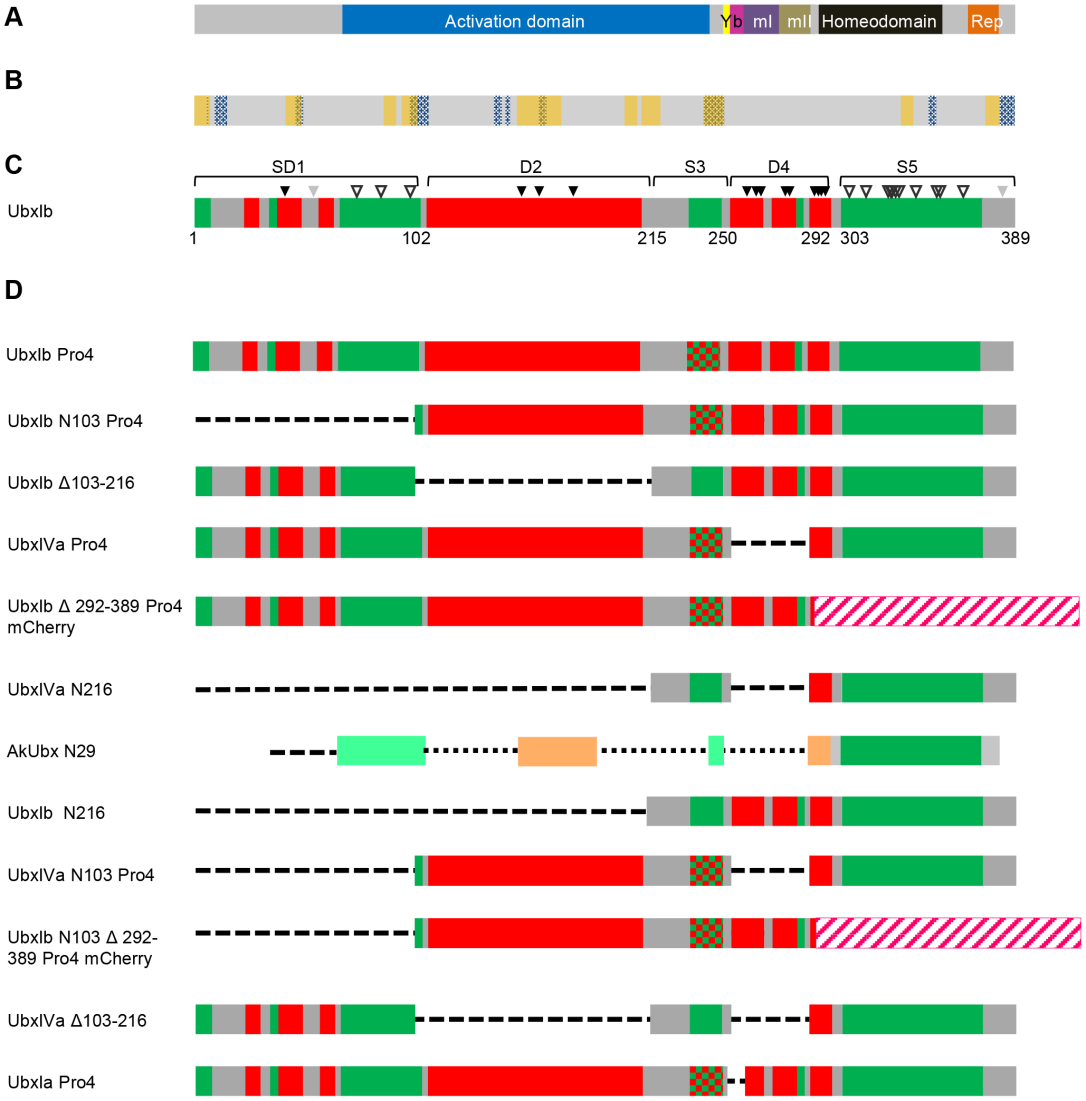
Location of structured and disordered regions in UbxIb, and design of Ubx variants. (A) A grey bar, representing the domain organization of the UbxIb transcription factor shows the position of its transcription activation domain (blue), YPWM Exd interaction motif (yellow), DNA-binding homeodomain (black), a partial transcription repression domain (orange), and protein regions encoded by three alternatively spliced microexons: the b element (pink), mI (purple), and mII (brown). (B) The location of predicted protein-interaction motifs in Ubx as predicted by ANCHOR (yellow stripes) and MoRFpred (blue stippled stripes). Regions predicted by both algorithms to be involved in protein interactions are marked with both yellow and blue. (C) A bar schematic depicting the positions of structured and intrinsically disordered regions in UbxIb. The boundaries were determined by a combination of computational and experimental approaches. The scores from three disorder prediction algorithms were averaged to identify structured (green) and disordered (red) regions. Native state proteolysis, in which only disordered segments can be cleaved by trypsin, was used to verify these assignments, and, where appropriate, slightly expanded the boundaries of the predicted disordered regions [6]. Sites cut by trypsin (black triangles), sites not cut by trypsin (open triangles), and sites that could not be definitively assigned (grey triangles) are indicated. (D) Bar schematic for predicted protein interfaces and molecular recognition features (MoRFs) on Ubx peptide. The schematic bars show Anchor algorithm predicted Ubx- partner protein interfaces (orange bars) and MoRF algorithm predicted Ubx-partner protein interface (blue bars with pattern fill). (D) Bar schematics of Ubx truncation mutants and internal deletion mutants used in yeast two-hybrid assays to identify partner binding interfaces. UbxIb, UbxIa, and UbxIVa are isoforms created by alternative splicing *in vivo*. To prevent auto-activation, the activation domain was de-activated either by removal of amino acids 102 to 216 or by the Pro4 mutation, in which Ala and Glu are mutated to Pro at amino acids 226 and 233 (indicated by a red-green stipple), respectively, which should prevent formation of a predicted α-helix required for transcription activation [43]. In two variants, the structured C-terminus of the protein was replaced by mCherry, represented by a pink/white striped bar.

In this paper, we explore the importance of partner topology in protein interactions mediated by Ultrabithorax (Ubx), a *Drosophila melanogaster* Hox transcription factor. Ubx is composed of both structured and disordered regions (Figure 1) [6], [42], [43]. Amino acids 1–102 of Ubx, herein termed Region 1, include a mixture of short structured elements interspersed with disordered sequences. Region 2 is a large disordered region, spanning amino acids 103 to 216 and including a portion of the transcription activation domain [43]. A putative α-helix required for transcription activation is located in Region 3 [43]. Amino acids 250–303, termed Region 4, encompass intrinsically disordered, alternatively spliced microexons and the disordered N-terminal arm of the homeodomain. Finally, the C-terminal Region 5 includes the structured portion of the homeodomain. Based on native state proteolysis rates, the disordered regions of Ubx are significantly more exposed than the disordered regions of proteins that fold upon ligand or co-factor binding [6]. Moreover, Region 2 is extremely glycine rich (27%, including 13 contiguous glycines). Polyglycine peptides are compact, yet very dynamic, and lack stable intraprotein contacts [44], [45]. Because the extent of monomer disorder correlates with the degree of disorder present in the bound state [34], [46], [47], the extremely dynamic disordered regions in Ubx are unlikely to fold into a stable structure upon partner protein binding.

Ubx is a “one-to-many” protein, in that it physically interacts with 39 known partner proteins with a wide variety of molecular functions [42], [48]–[54]. This large number of partner proteins provides a sufficiently diverse sample to identify common traits that enable binding to Ubx. Several of these interactions have been validated *in vivo* [48]–[50]. Proteins that genetically interact with Ubx, unsupported by physical interaction data, were not included in this study since genetic interactions can arise from processes other than physical interaction between proteins. We found that specific folds are significantly enriched in Ubx-interacting proteins. Single domains of the partner protein that exhibit the selected fold are sufficient to bind Ubx. Interestingly, the intrinsically disordered regions of Ubx are necessary for these protein interactions. Although partners bind all three disordered regions cooperatively, individual partner proteins rely on specific disordered regions to varying extents, creating opportunities for competition and collaboration in forming higher order complexes. Regions 1 and 2 are multiply phosphorylated, providing another mechanism to regulate partner binding *in vivo*. Partner binding also varies among Ubx isoforms arising from *ubx* mRNA splicing, providing a third regulatory mechanism. Interestingly, the preference of protein partners for specific Ubx isoforms correlates well with the topology of the partner protein. Thus, phosphorylation and alternative splicing, both tissue-specific processes, have the potential to regulate protein interactions. The regions of Ubx involved in partner binding also regulate DNA binding and include a portion of the transcription activation domain [6], [7], [43]. Linking different Ubx functions via intrinsically disordered regions has the potential to provide the specificity and reliability required for Hox activity *in vivo*.

## Materials and Methods

### Definition of Intrinsically Disordered Regions of Ubx

Ubx disordered regions were defined by a combination of prediction algorithms and experimental assays. Disordered and structured regions were predicted using the average score from three programs, VLXT-PONDR, IUPRed, and DisEMBL (loops/coils) [6]. Predicted amino acid residues with an average prediction score ≥0.6 are designated disordered. A residue with an averaged prediction score between 0.4 and 0.6 was considered as uncertain and thus was not defined in this study. A residue with an average prediction score ≤0.4 was considered structured. Native state proteolysis data [6] were used to refine the predicted boundaries of disordered and structured regions. Since successful proteolysis requires a minimum of five disordered amino acids on both sides of the severed bond, the regions designated as disordered were expanded at a few positions to include these sequences. The designations of structure and disorder agree with previous data on the locations of structure in the Ubx homeodomain, the partially structured nature of the HoxB1 FPWM motif in the absence of Pbx1 binding (analogous to the Ubx YPWM motif which was designated as “uncertain” by our analysis), and the location of a putative α-helix involved in transcription activation by Ubx [42], [43], [55]. Protein interface and molecular recognition features were predicted by the Anchor and MoRFpred algorithms, respectively [52]–[54].

### Classification of the *Drosophila* Interactome by Fold

The *Drosophila melanogaster* large-scale yeast two-hybrid dataset [50] was used for this global analysis. The structural assignments, definitions, and evolutionary relationships listed in Flybase [56] and the Structural Classification of Proteins (SCOP) database version 1.65 release 3 [57] were used to group the proteins by folds. SCOP merges computer algorithms and human curation to classify protein domains based on structural and evolutionary similarities. Interaction maps were generated and modified using Osprey 1.20 (http://biodata.mshri.on.ca/osprey/servlet/Index).

Databases built using Microsoft Access were used to construct the figures and tables in the Supporting Data, which can be accessed from http://rice.allgeek.net. Algorithms to analyze the raw protein interaction data were written using Windows Visual Basic 6.0. The genome database was compiled from a list of all *Drosophila* genes downloaded from Flybase. If the Flybase reference for the corresponding protein had one or more assigned folds as defined by SCOP, then all potential fold-fold pairs were included in the database. Any structure assignments that were fragments of other folds, “not a true fold”, or duplicates of other entries were eliminated. By this analysis, roughly one quarter of *Drosophila* proteins have an assigned fold. Each fold in multifold proteins was included in the genome database, accounting for 23% of the proteins, and was listed as an interacting fold for all interactions in which the multifold protein participates, yielding 63% of the total interactions examined.

The interactome database contains previously defined interactions and includes the confidence score assigned to that interaction by Giot *et al.* [51]. Data fitting for the scale-free graph was completed using Igor Pro Version 4.02A (WaveMetrics).

### Classification of Ubx protein interactions by fold

Proteins with assigned folds that physically interact with Ubx included data from Giot *et al.* [51], our laboratory [48], [49], and other laboratories [50]. Proteins encoded by genes that only genetically interact with *ubx* were not included, because molecular events other than protein interactions can yield a genetic interaction. Folds within this protein list were identified as described above.

### Yeast two-hybrid assays

Ubx deletion and truncation mutants were created using the QuikChange site-directed mutagenesis kit following manufacturer instructions (Agilent). Ubx variants were cloned into the pLexA plasmid (Clontech) between the EcoRI and BamHI restriction enzyme sites. Ubx binding partners had previously been cloned into the pB42 vector [48], [49]. DNA encoding the individual domains of Al (residues 81 to 142) and Arm (residues 155 to 273) were synthesized by Blue Heron Biotechnology Inc., USA.

Ubx variants and partner plasmids were co-transformed into EGY48 *Saccharomyces cerevisiae* already carrying the p8op-LacZ reporter plasmid (Clontech). In this process, 500 µl of an overnight liquid culture of yeast (OD_600 nm_≈1.5) was centrifuged, and the pellet was washed with 2 mM lithium acetate (Acros) and 100 mM dithiothreitol (DTT, Fisher Scientific). Cell pellets, resuspended in 100 µl of transformation reaction mix, containing 2 mM lithium acetate, 50% polyethylene glycol (Sigma, MW3350), 10 µg/ml salmon sperm DNA (Sigma), and 100 mM DTT, were mixed with Ubx-pLexA plasmid and Ubx binding partner pLexA fusion (500 ng per plasmid). The resulting mixture was incubated at 46°C for one hour and subsequently centrifuged. The pellet was re-suspended in sterile water and spread on a designated synthetic amino acids drop-out yeast medium agar (2%) plate containing 80 µg/ml X-gal (Research Products International) following incubation for 5–6 days at 30°C.

The blue or white color of the colonies provided an initial qualitative measure of binding. The results of this qualitative assay matched subsequent quantitative results using the Miller β-galactosidase reporter assay [58], [59]. In this assay, an individual yeast colony was used to inoculate 5 ml of the designated synthetic amino acid drop-out yeast medium, then grown overnight at 30°C with 250 rpm shaking to an OD_600 nm_≈1.5. β-Galactosidase liquid assays generally followed the Clontech Yeast Protocols Handbook (Clontech). In brief, 2 ml of the overnight yeast culture were used to inoculate 8 ml of the trp^−^/his^−^/ura^−^ drop-out yeast medium containing 10% galactose (Sigma) to activate the B42-partner chimera and 5% raffinose (Sigma) to provide a carbon source and incubated at 30°C for 3–5 hr with 250 rpm shaking until the cells reached mid-log phase with OD_600 nm_≈0.8. To harvest the yeast culture, 1.5 ml was removed and centrifuged 10,000×*g* for 30 seconds. Supernatant was discarded and the pellet was mixed thoroughly with 1.5 ml of Z Buffer (70 mM Na_2_HPO_4_, 40 mM NaH_2_PO_4_• H_2_O, 10 mM KCl, 1.3 mM MgSO_4_). After re-centrifugation and decanting the supernatant, the pellet was resuspended in 300 µl of Z Buffer, divided into three 100 µl aliquots, frozen in liquid nitrogen for 1 minute, and incubated at 37°C for 45 seconds. This freeze and thaw process was repeated two more times. To the cell lysate, 4 mg/ml of *ortho*-nitrophenyl-β-galactoside (ONPG, Sigma) in Z Buffer and 700 µl of 27% β-mercaptoethanol in Z buffer were added, followed by 30°C incubation with mixing by inversion every 10 minutes. β-Galactosidase expression levels were assessed by enzymatic assays that spectroscopically measure generation of the β-galactosidase enzymatic product, *o*-nitrophenol (ONP), at 420 nm. When yellow color was visible, reactions were quenched by addition of 400 µl of 1 M Na_2_CO_3_. The elapsed time from the beginning of the reaction (ONPG addition) to the end of reaction (Na_2_CO_3_ addition) was recorded. The reaction mixture was centrifuged at 10,000×*g* for 10 minutes. Supernatant was collected and A_420 nm_ was recorded. The results were reported in Miller units, the amount of β-galactosidase that hydrolyzes 1 µmol of ONPG to ONP per min per cell [58], [59]. Miller units were calculated using the following formula: 
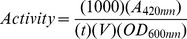
(Eqtn \kern 1 1)in which *t* is the elapsed time (in min) of incubation, *V* is 0.1 ml× dilution factor (5 for this protocol), *OD_600 nm_* is the optical density of 1 ml induction culture before harvest measured at a wavelength of 600 nm, and *A_420 nm_* is absorbance of 1 ml ONPG reaction product measured at 420 nm.

### Western Blotting

Extraction of yeast protein samples and their preparation for western blotting followed the Yeast Protocols Handbook (Clontech). Cells were lysed as described for yeast two-hybrid assays, and whole cell lysate was subsequently centrifuged at 10,000×*g* for 10 minutes to remove cell debris and any insoluble Ubx. Proteins were separated by SDS-PAGE prior to western blotting with a 1∶200 dilution of LexA murine monoclonal primary antibody (Santa Cruz Biotechnology) followed by a 1∶5000 dilution of IRDye 800CW Goat anti-Mouse IgG (H+L) secondary antibody (Li-Cor). Protein expression was visualized and quantified using an Odyssey infrared imaging system and software (Li-Cor).

## Results

### Ubx selects protein interactions based on partner topology

The *Drosophila* Hox protein Ubx is 44% intrinsically disordered, and binds many partner proteins [48]–[50], [60]. However, the location and chemical nature of most of these protein interfaces is unknown. To determine which physicochemical properties of partner proteins are important for mediating these interactions, we first examined the characteristics of Ubx-binding proteins (Figure 1). Although some of the Ubx partner proteins form true interactions that alter Ubx function *in vivo* [47]–[49], other interactions have not been examined in flies. In addition, a few interactions are unlikely to be biologically relevant because the partner has a different sub-cellular localization and/or is involved in unrelated biological processes [49]. However, binding by all partners results in similar reporter intensities in yeast two-hybrid assays, reflecting similar protein interaction affinities. Ubx is presumably able to bind the unlikely partners *in vitro* because these proteins share features recognized by Ubx when it binds the true partners. Therefore, including these unlikely partners in the analysis increases the occurrence of traits selected by Ubx while simultaneously decreasing the occurrence of traits commonly found in transcription factors but not specifically required for Ubx binding.

**Figure 2:**
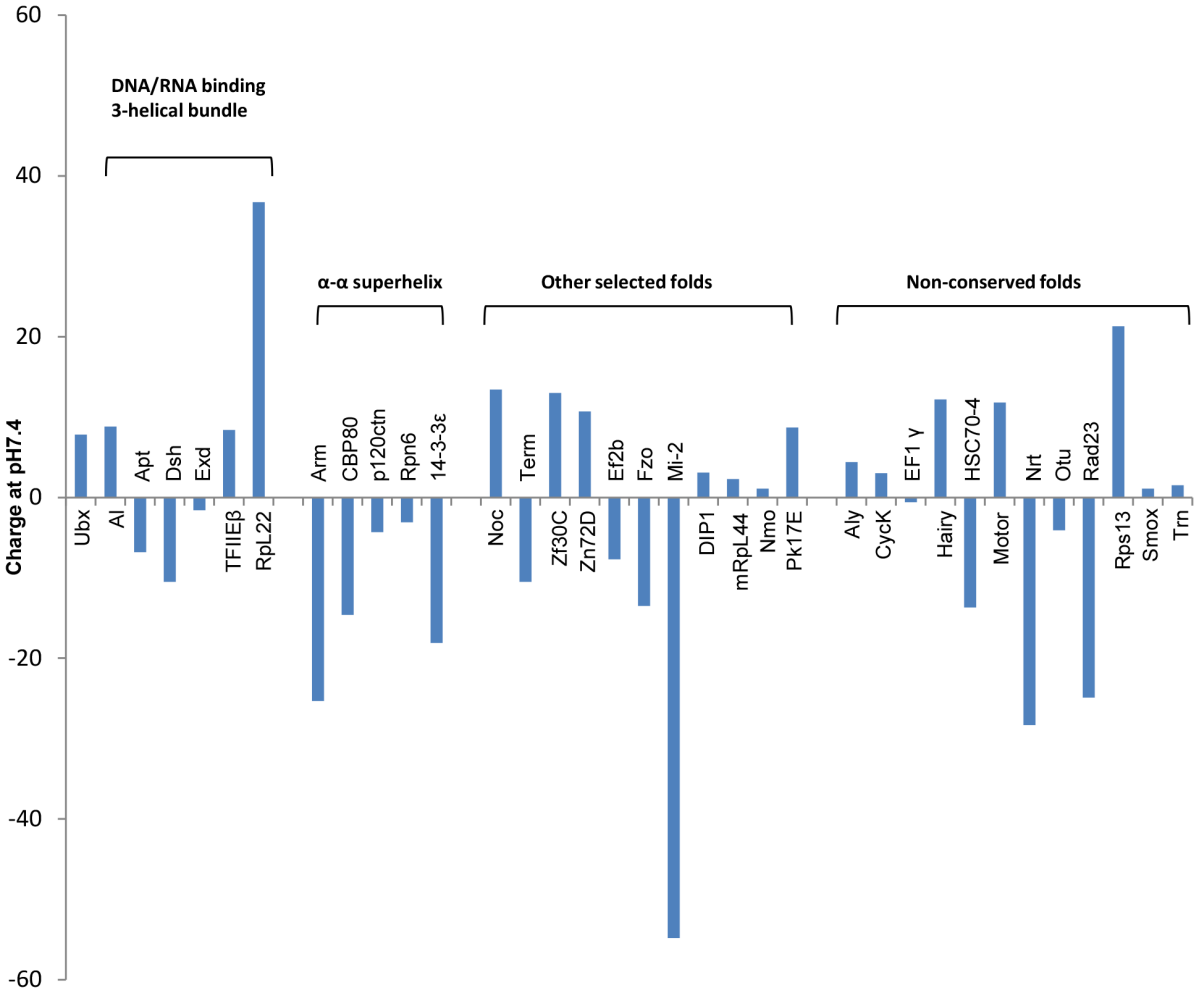
Ubx binds both positively and negatively charged proteins. The chart shows the predicted net charge at pH = 7.4 of Ubx and the subset of its partner proteins with known folds [48]–[50]. Abbreviations: Al, Aristaless; Aly, Always early; Apt, Apontic; Arm, Armadillo; CycK, Cyclin K; CBP80, Cap-binding protein 1; Dsh, Dishevelled; DIP1, Disconnected-interacting protein 1; Ef2b, Elongation factor 2b; EF1 γ, Elongation factor 1γ; Exd, Extradenticle; Fzo, Fuzzy onions; mRpL44, Mitochondrial ribosomal protein L44; HSC70-4, Heat shock protein cognate 4; Nmo, Nemo; Noc, No ocelli; Nrt, Neurotactin; p120ctn, Adherens junction protein p120; Otu, ovarian tumor; PK17E, Protein kinase-like 17E; RpL22, Ribosomal protein L22; Rpn6, Protease p44.5 subunit; Rps 13, Ribosomal protein S13; Smox, Smad on X; Term, terminus; Trn, Transportin; TFIIEβ, Transcription factor IIEβ; Ubx, Ultrabithorax; Zf30C, Zinc finger protein 30; Zn72D, Zinc-finger protein at 72D.

Of the 39 known Ubx binding proteins [42], [48]–[54], 34 have domains with assigned folds. We searched for properties common to these 34 Ubx partners. Ubx has a predicted net charge of +7.3 at pH = 7.4. This strong positive charge is largely due to the DNA-binding homeodomain (+11 at pH = 7.4), the only large structured region within Ubx. Any proteins directly binding the homeodomain would be expected to have a compensating negative charge. Ubx partners have a surprisingly large range of predicted net charges at pH = 7.4, spanning +36 to −54 (Figure 2). Thus, net charge does not correlate with the ability to bind Ubx, suggesting that all partners are unlikely to exclusively bind the positively charged homeodomain.

Topology is a key factor affecting interactions between structured proteins, and sorting protein interactions based on the folds of the interacting partners can yield useful information about the nature of the interactions [41]. Using the terminology of the Structural Classification of Proteins (SCOP) hierarchical classification database [57], [61], analysis of Ubx partners at the level of protein folds reveals that 23 of the 34 Ubx binding partners contain one of just 7 different folds, out of the 1195 folds identified by SCOP (Table 1, Table S1). All of the selected folds in Ubx-interacting proteins are enriched relative to the frequency with which these folds occur in the *Drosophila* proteome (Table 2). However, this level of enrichment may not be specific to Ubx: some folds are more prevalent in the *Drosophila* interactome. To determine whether these folds are more likely to bind Ubx than a random protein, we compared the extent of fold enrichment among Ubx partners with data derived from a high-throughput yeast two-hybrid experiment on *Drosophila* proteins [51] (Figure S1). Grouping the high-throughput data by fold did not change the scale-free nature of the network (Figure S2). The DNA/RNA binding 3-helical bundle fold, the α-α superhelix fold, and the dsRNA binding motif fold occur more frequently among Ubx-interacting proteins than in the *Drosophila* interactome, indicating that the enrichment of these folds among Ubx partners is not an artifact of their increased propensities to bind proteins in general (Table 2). For Ubx and each protein in the *Drosophila* interactome, we also calculated the number of folds each protein binds (F) divided by the number of proteins each binds (I) (Figure S3). Proteins with an F/I ratio approaching 1 do not select partners by topology, whereas proteins with a low F/I ratio are highly selective. Whereas Ubx has an F/I ratio of 0.61, approximately 90% of the proteins analyzed have a higher F/I ratio, indicating they are less selective than Ubx. Despite the fact that large regions within Ubx are disordered and presumably extremely dynamic, these results suggest that topology is an important criterion by which Ubx selects protein partners.

**Table 1:** Specific folds are enriched in Ubx-binding proteins.

Fold	Partner	Fold	Partner
**DNA/RNA binding 3-helical bundle**	RpL22	**P-loop containing NTP hydrolases**	EF2b
	Apt		Mi-2
	Al		Fzo
	Dsh		
	Ubx	**dsRBD-like**	DIP1
	Exd		mRpL44
**α-α superhelix**	Arm	**Ferridoxin-like**	EF2b
	Rpn6		Aly
	P120ctn		
	CBP80	**Protein kinase-like**	Nmo
	14-3-3ε		Pk17E
**Zinc-Finger C2H2 and C2HC**	Noc		
	Zf30C		
	Zn72D		
	Term		

Exd is a well-established Ubx binding protein [50], and Ubx cooperatively binds DNA [77]. All other Ubx binding partners were identified by yeast two-hybrid assays. Ubx binding partners were classified by the fold/shape according to SCOP. Folds with more than one partner were defined as “selected”. The interactions with Term, Fzo, mRpL44, and Pk17E were reported by Giot *et al.* [51]. The remaining interactions were reported by Bondos *et al.* [48], [49]. Ubx binding proteins with non-selected folds are listed in Table S1.

**Table 2:** A comparison of the occurrence of folds in the *Drosophila* proteome and interactome.

Fold	Frequency in *Drosophila* proteome	Frequency in *Drosophila* interactome	Frequency in Ubx partner list	P-value of enriched fold relative to *Drosophila* proteome	P-value of enriched fold relative to *Drosophila* interactome
DNA/RNA binding 3-helical bundle	2.7%	8.4%	17.6%	P<0.05	0.05<P<0.1
α-α superhelix	3.4%	7.2%	14.7%	P<0.05	0.05<P<0.1
Zinc Finger C2H2 and C2HC	3.7%	11.7%	11.8%	P<0.05	0.05<P
dsRBD-like	0.2%	0.9%	5.9%	P<0.05	P<0.05
Protein kinase-like	2.8%	5.6%	5.9%	0.05<P	0.05<P
p-loop containing NTP hydrolases	5.6%	8.4%	8.8%	0.05<P	0.05<P
Ferridoxin-like	2.8%	8.1%	5.9%	0.05<P	0.05<P

p-value of enriched fold relative to *Drosophila* proteome/interactome was generated using Chi-Squared test.

Importantly, proteins unlikely to naturally bind Ubx can have the same fold as true Ubx partners. For example, DIP1 alters transcription regulation by Ubx in cell culture assays and inhibits Ubx function *in vivo* [48]. DIP1 has the same fold as mRpL44, a mitochondrial ribosomal protein that should not co-localize with Ubx *in vivo*. Therefore, even if some Ubx-protein interactions lack a biological role, they can still yield information regarding the physicochemical properties of partner proteins bound by Ubx *in vivo*. This phenomenon underscores the importance of partner topology in the selection of protein partners by Ubx.

**Figure 3:**
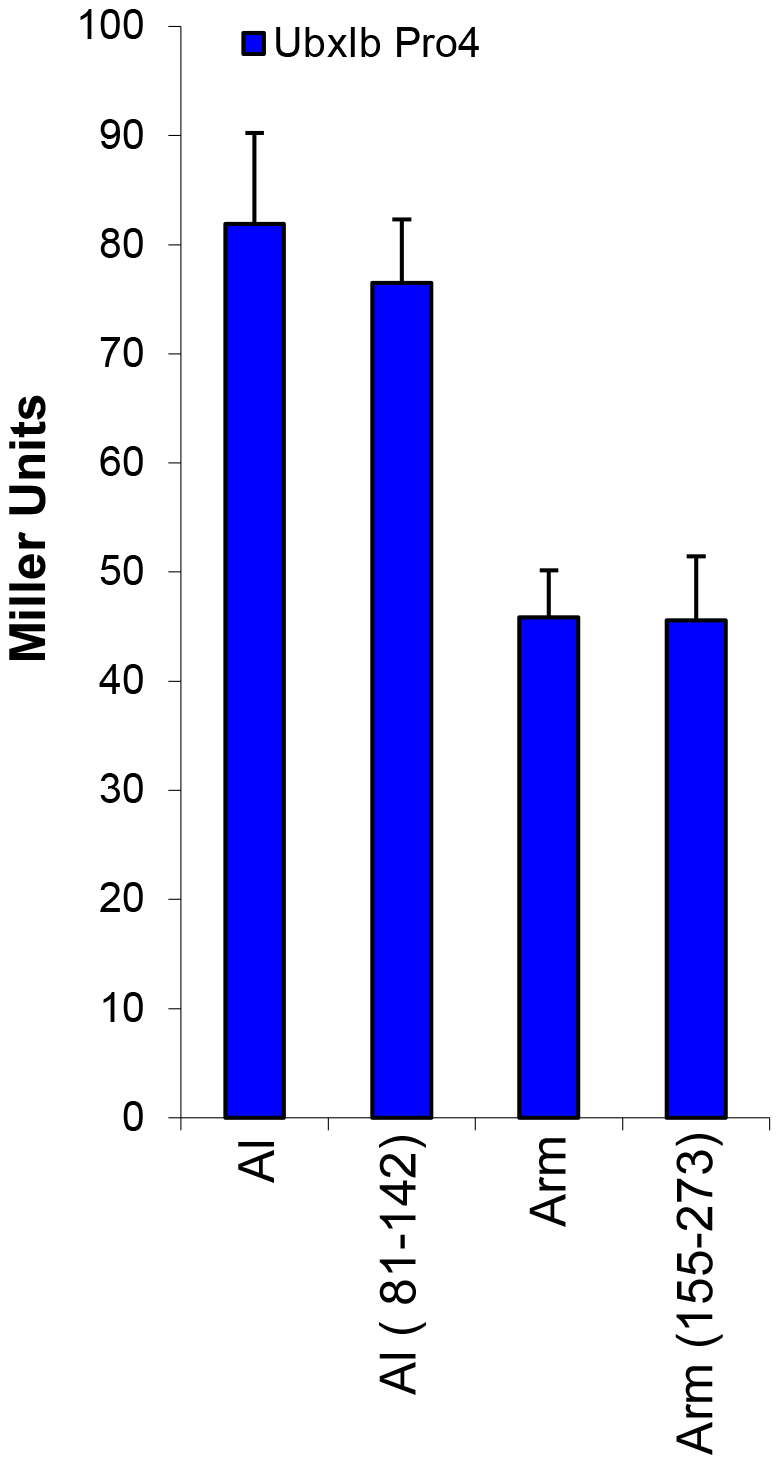
An individual partner domain is sufficient for Ubx binding. Full length Al and Arm have similar interaction strength as individual domains derived from Al (residues 81–142) and Arm (residues 155–273) with UbxIb Pro4. The intensity of the β-galactosidase reporter gene, reported as Miller Units, signal for each partner is similar to its respective single-domain variant.

The enrichment of particular folds among Ubx partners may be caused by Ubx preferring to bind the surface topologies created by these folds. Alternately, the types of proteins Ubx binds *in vivo*, transcription factors and cell signaling proteins, may be enriched in these folds (*e.g.,* a DNA/RNA binding 3-helical bundle fold). Consequently, the “selected folds” may be enriched among Ubx partners due to their cellular function rather than presentation of a binding interface on the surface of the selected fold. In order to determine whether the selected folds are sufficient to mediate Ubx interactions, we used yeast two-hybrid assays to probe whether Ubx interacts with the regions of partner proteins that correspond to the selected topology. We utilized the yeast-two hybrid method because (i) these assays do not interfere with Ubx binding to these partners, (ii) these assays do not rely on other Ubx functions, such as DNA binding or transcriptional regulation, iii) yeast two-hybrid assays allow quantitative comparison of the strength of binding, and (iv) many partners identified by yeast two-hybrid assays also alter Ubx function *in vivo* [48]–[50], [62], demonstrating this method likely reflects native protein interactions involving Ubx. We created two constructs: a single α-α superhelix domain from Arm (amino acids 155–273) and a DNA/RNA binding 3-helical bundle domain from Al (amino acids 81–142). We hypothesized that the individual domain in a Ubx partner is sufficient to interact with full-length Ubx without surrounding sequences. To prevent reporter gene activation by Ubx in the absence of partner binding, a full-length Ubx mutant (UbxIb Pro4) was used that is incapable of transcription activation [43]. Individual yeast two-hybrid experiments between these two isolated domains and UbxIb Pro4 [43], exhibit similar levels of reporter gene expression as for experiments in which UbxIb Pro4 binds the corresponding full-length partners (Figure 3). This result indicates that the α-α superhelix x and DNA/RNA binding 3-helical bundle folds in these proteins are sufficient for Ubx interaction.

### More than one region of Ubx is required for protein interactions

Ubx contains both structured and intrinsically disordered domains, either of which could mediate protein interactions and potentially select partners based on topology. One important function of intrinsically disordered regions is to mediate protein interactions [14]–[24], suggesting the disordered regions in Ubx may serve as or contribute to protein interaction domains. Conversely, fold selection is a known property of interactions between structured proteins [12], [13] and has not been previously observed for intrinsically disordered proteins, suggesting Ubx partners may bind the structured regions of Ubx. We tested whether the structured or disordered regions of Ubx mediate binding using yeast two-hybrid assays. We focused our studies on the folds that include the greatest number of Ubx-interacting proteins: the α-α superhelix fold (5 proteins) and the DNA/RNA binding 3-helical bundle fold (6 proteins). This selection of multiple partners optimizes the possibility of identifying characteristics important for Ubx binding.

Our general approach is to remove either intrinsically disordered or structured sequences within Ubx (Figure 1) to assess whether these regions impact binding to protein partners. All of the Ubx mutants were carefully designed to minimize the impact on regions of Ubx structure that are well-folded. In the N216 and N103 Ubx truncation mutants, amino acids 2–215 (Regions 1 and 2) or 2–102 (Region 1) are removed, respectively (Figure 1). These variants have been successfully used for both *in vitro* DNA binding assays and yeast one- and two-hybrid experiments [6], [43]. Indeed, both truncation mutants are soluble, active monomers capable of binding DNA with an affinity comparable to full-length Ubx [6]. Similarly, we made Ubx variants with an internal deletion (Δ103-216) which removes Region 2. Other Ubx mutants with internal deletions in this region are also soluble and capable of binding DNA [6]. Furthermore, the length of this region is significantly reduced in natural Ubx orthologues (Figure S4) [63], consistent with observations that this internal deletion in *Drosophila* Ubx does not significantly disrupt the remaining Ubx structure [6]. The C-terminal disordered region (Region 4) spans an alternatively spliced region of Ubx. The natural Ubx spliceoform UbxIVa removes nearly all (90%) of the intrinsic disorder in this region, and was used to assess the contribution of Region 4 to protein interactions.

**Figure 4:**
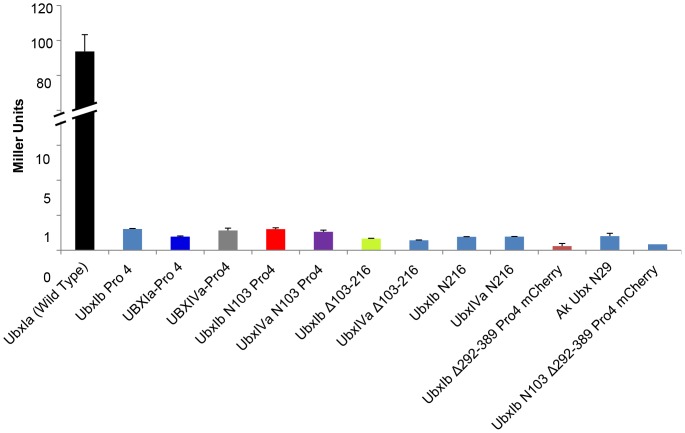
Ubx variants did not interact with B42 protein activation in the absence of Ubx partners. Yeast two-hybrid results for wild type full length Ubx or Ubx variants with truncation and/or Pro4 mutation showed no significant interaction with B42 protein activation domain from β-galactosidase reporter gene expression, listed as Miller Units.

Because Ubx is fused to the LexA DNA-binding domain in the yeast two-hybrid assay, the transcription activation domain in Ubx was deactivated in each mutant to prevent the LexA-Ubx fusion from activating the reporter gene and generating false positive signals. This deactivation was accomplished either by removing a critical portion of the activation domain (amino acids 103–216) or by including the mutations A226P/Q233P, abbreviated as “Pro4”, to unfold a putative α-helix required for transcription activation [43]. None of the Ubx variants in this study were able to activate transcription on their own, or bind products of the empty bait vector pB42 (Figure 4). Furthermore, the expression levels of all Ubx variants in yeast were similar, except the two Ubx fusion proteins in which the DNA-binding homeodomain was replaced with mCherry, which were expressed at much higher levels (Figure S5).

To clarify which portions of the Ubx sequence are included or removed in each variant, the name of each Ubx variant in this text is introduced followed by a notation representing the Ubx sequences present in parentheses. We have divided the Ubx sequence into 5 regions (Figure 1). The number representing each region will be preceded by an S if the region is structured, a D if the region is disordered, and SD if that region contains both structured and disordered elements. Thus the sequence of full-length, wild-type Ubx would be depicted as (SD1, D2, S3, D4, S5). Regions that are missing or mutated in a particular variant are designated by 0. The UbxIb Pro4 mutant, in which the helix in region S3 has been destabilized by mutation to prevent transcription activation, would be notated as (SD1, D2,0, D4, S5).

**Figure 5:**
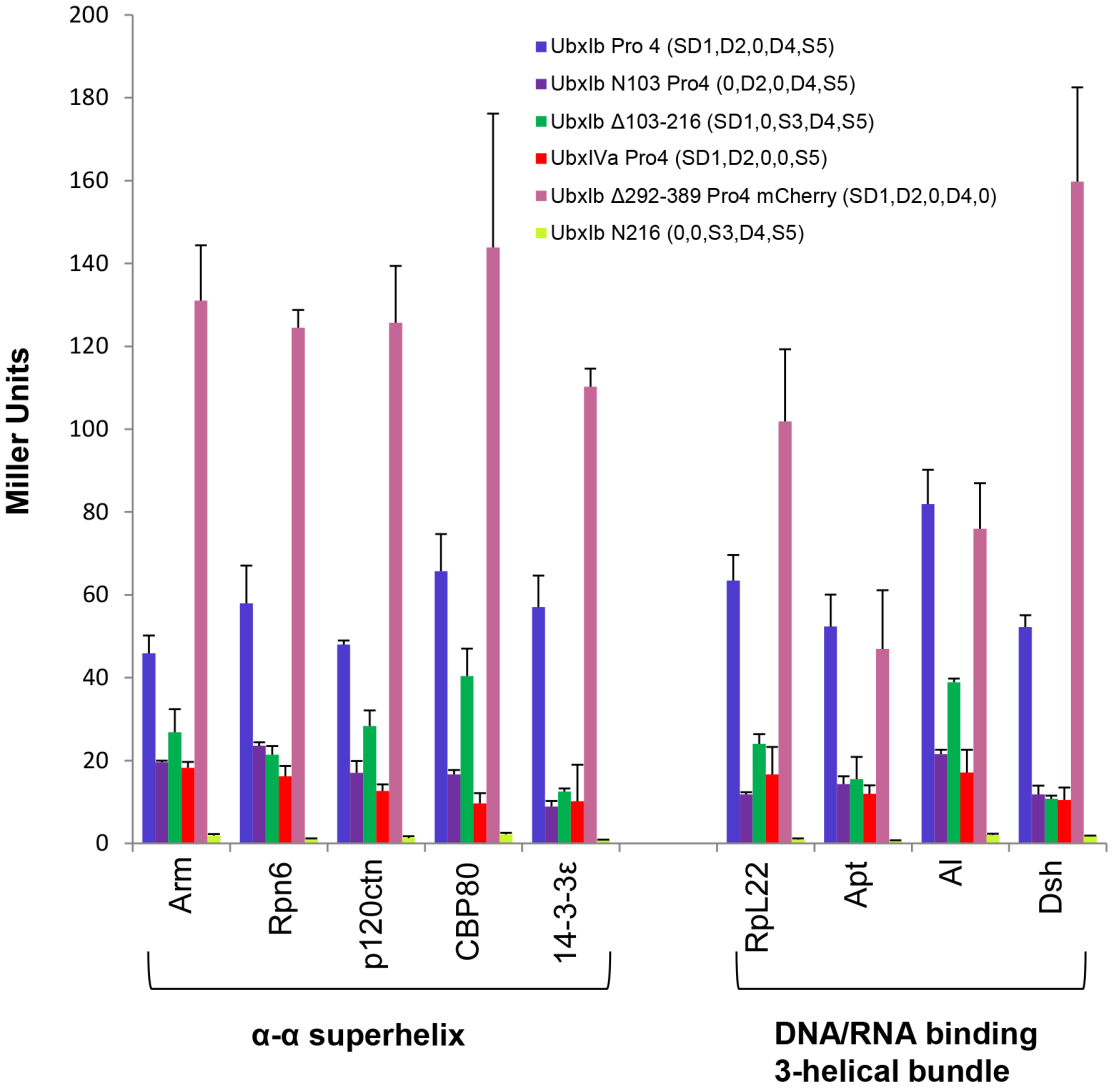
Partner proteins bind more than one region in Ubx. Yeast two-hybrid results for Ubx variants in which each region of Ubx has been sequentially mutated or deleted. Each of these variants retain some ability to bind Ubx relative to UbxIbN216 (0,0, S3, D4, S5). Partners are grouped based on the fold they have in common.

We made a series of Ubx truncations or mutations to sequentially test whether each portion of the Ubx sequence contains a critical partner binding site (Figure 5). All data were compared with UbxIb Pro4 (SD1, D2,0, D4, S5), a full-length variant of Ubx which binds all partners but cannot activate the reporter gene in the absence of partner interaction. UbxIb N103 Pro4 (0, D2,0, D4, S5), in which the structured and disordered elements in Region 1 were removed, still bound the partner proteins, indicating Region 1 is dispensable for partner binding. UbxIb Δ103–216 (SD1,0, S3, D4, S5), which removes the intrinsically disordered Region 2, also bound some partners. The previously established ability of UbxIb with the Pro4 mutation (SD1, D2,0, D4, S5) to bind partners indicates that the helix in Region 3 cannot be responsible for partner binding [48], [49]. Conversely, the Pro4 mutations are not required for partner binding, because partners bind UbxIb Δ103–216 (SD1,0, S3, D4, S5), which retains the wild-type helix sequence in Region 3. UbxIVa Pro4 (SD1, D2,0,0, S5) binds partner proteins, even though the disordered Region 4 has been removed. Finally we created UbxIb Δ292–389 Pro4 mCherry (SD1, D2,0, D4,0), in which the structured C-terminus (Region 5) has been removed and replaced with the mCherry protein sequence. mCherry alone is unable to bind any of the Ubx partners (data not shown). However, UbxIb Δ292–389 Pro4 mCherry bound all partners, indicating that Region 5, which includes the DNA-binding homeodomain, is not necessary for partner binding. Collectively, these data indicate that more than one region of Ubx is required for protein interactions.

### The intrinsically disordered regions in Ubx are necessary for protein interactions

**Figure 6:**
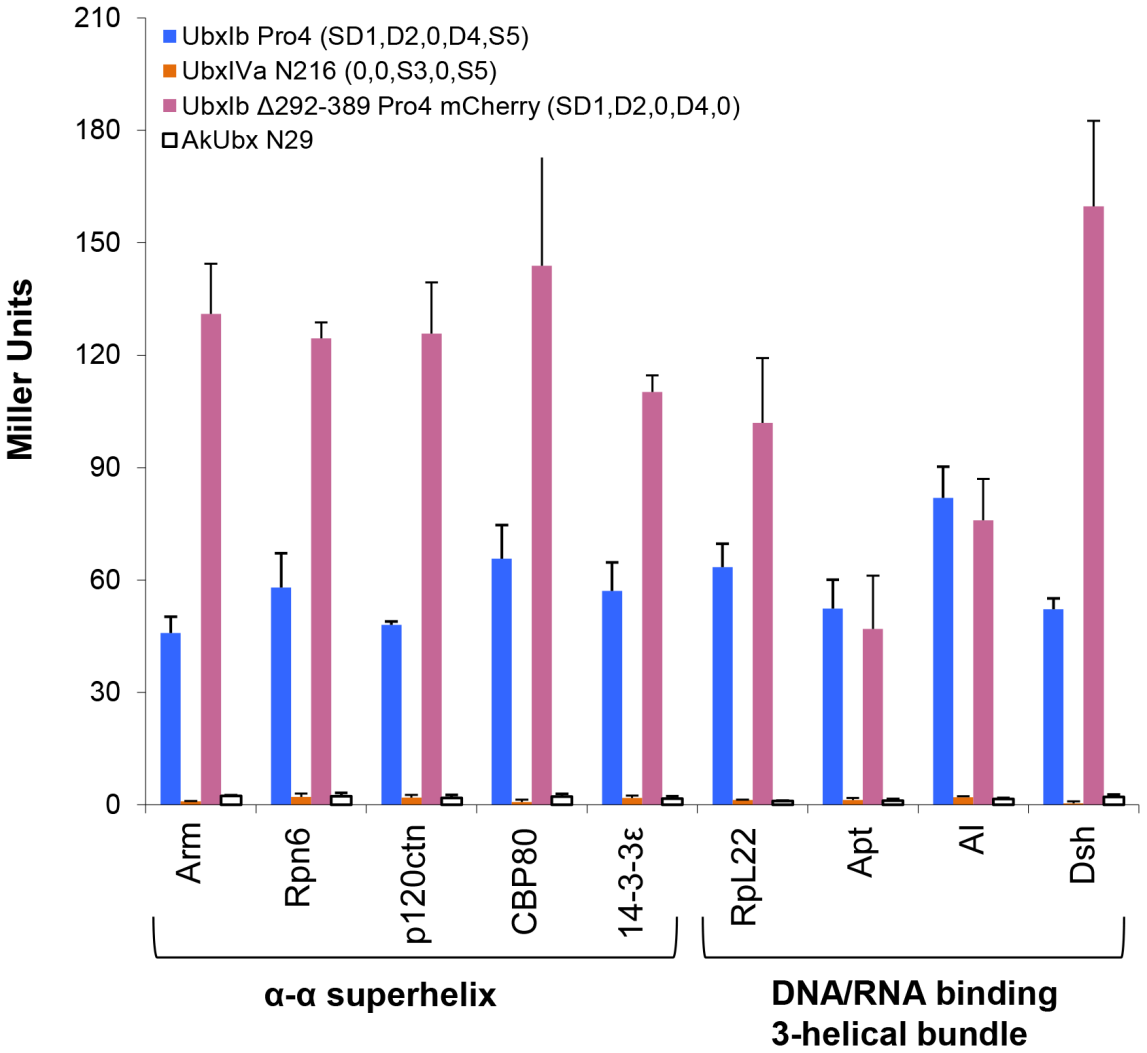
The intrinsically disordered regions in Ubx are necessary for protein interactions. Yeast two-hybrid indicates that Ubx variants, either lacking all disordered regions (UbxIVa N216) or all structured regions (UbxIb N103 Δ292–389 Pro4 mCherry), cannot bind partner proteins. Likewise, AkUbx, a primitive Ubx orthologue derived from *Acanthokara kaputensis*, naturally lacks most of the disordered sequences and is also unable to bind partner proteins.

The next step was to identify some portion of the Ubx protein that is necessary for partner binding. The MORF and ANCHOR algorithms both identify many short motifs in the intrinsically disordered regions of Ubx that have the potential to engage in protein interactions (Figure 1B), suggesting the intrinsically disordered regions may be collectively required for partner binding. In order to test this hypothesis, we compared binding by the structured versus disordered halves of the Ubx sequence. UbxIVa N216 (0,0, S3,0, S5) lacks all of the intrinsically disordered regions but retains two of the three regions containing structure. This mutant is based on the natural UbxIVa mRNA splicing isoform, which removes Region 3, and the N216 truncation, which removes Regions 1 and 2 (Figure 1D). The remainder of this Ubx variant is almost entirely structured (>90%). Conversely, UbxIb Δ292–389 Pro4 mCherry (SD1, D2,0, D4,0) retains all of the disordered regions, but lacks the Region 3 helix and the structured homeodomain in Region 5. UbxIVa N216 (0,0, S3,0, S5), which lacks intrinsically disordered sequences, was unable to bind all partner proteins, whereas UbxIb Δ292–389 Pro4 mCherry (SD1, D2,0, D4,0), which contains all of the intrinsically disordered sequences, bound all partners. In fact, this variant yielded an even more intense reporter signal than Ubx alone. Much of this elevated signal can be attributed to the increased expression level of UbxIb Δ292–389 Pro4 mCherry relative to the Ubx variants lacking mCherry (Figure 6). Thus Regions 1, 2, and 4, which include all of the intrinsically disordered regions in Ubx, are sufficient for partner binding.

One concern is that the structured regions may contribute to binding in the full-length protein, but are mis-positioned by the absence of the disordered regions in the UbxIVaN216 (0,0, S3,0, S5) mutant. To test the latter possibility, we examined whether the Ubx partners could bind an orthologue of Ubx derived from the velvet worm *Akanthokara kaputensis* (AkUbx), an onychorphoran whose last common ancestor with *Drosophila* lived 540 million years ago. Hox proteins in this ancient organism only have very basic molecular functions, which are reflected in the relatively simple and repetitive body plan of the animal [63], [64]. When expressed in *Drosophila*, AkUbx can replicate some, but not all, of the functions of *Drosophila* Ubx. Alignment of the Ubx and AkUbx sequences demonstrates that the disordered sequences in Regions 1 and 3 are absent in this ancient Ubx orthologue, and roughly half of the disordered sequences in Region 2 are missing (Figure S4). In contrast, the homeodomain and much of the structured portions of Region 1 are preserved. Therefore, by testing whether AkUbx can bind Ubx partners, we can use a native, folded Ubx orthologue to observe whether the loss of most of the intrinsically disordered regions prevents partner interaction. AkUbx showed little to no interaction with Ubx partners in the yeast two-hybrid assay (Figure 6). These results confirm that the disordered regions in Ubx are required for partner binding. Because no individual disordered region is solely responsible for partner interactions, we conclude that the intrinsically disordered regions in Ubx must cooperate to bind partner proteins. The requirement of multiple, non-contiguous disordered regions for partner interactions has been observed previously for other proteins [25], [39], [65].

### Either Region 1 or Region 4 is required as a scaffold to position intrinsically disordered Ubx sequences

**Figure 7:**
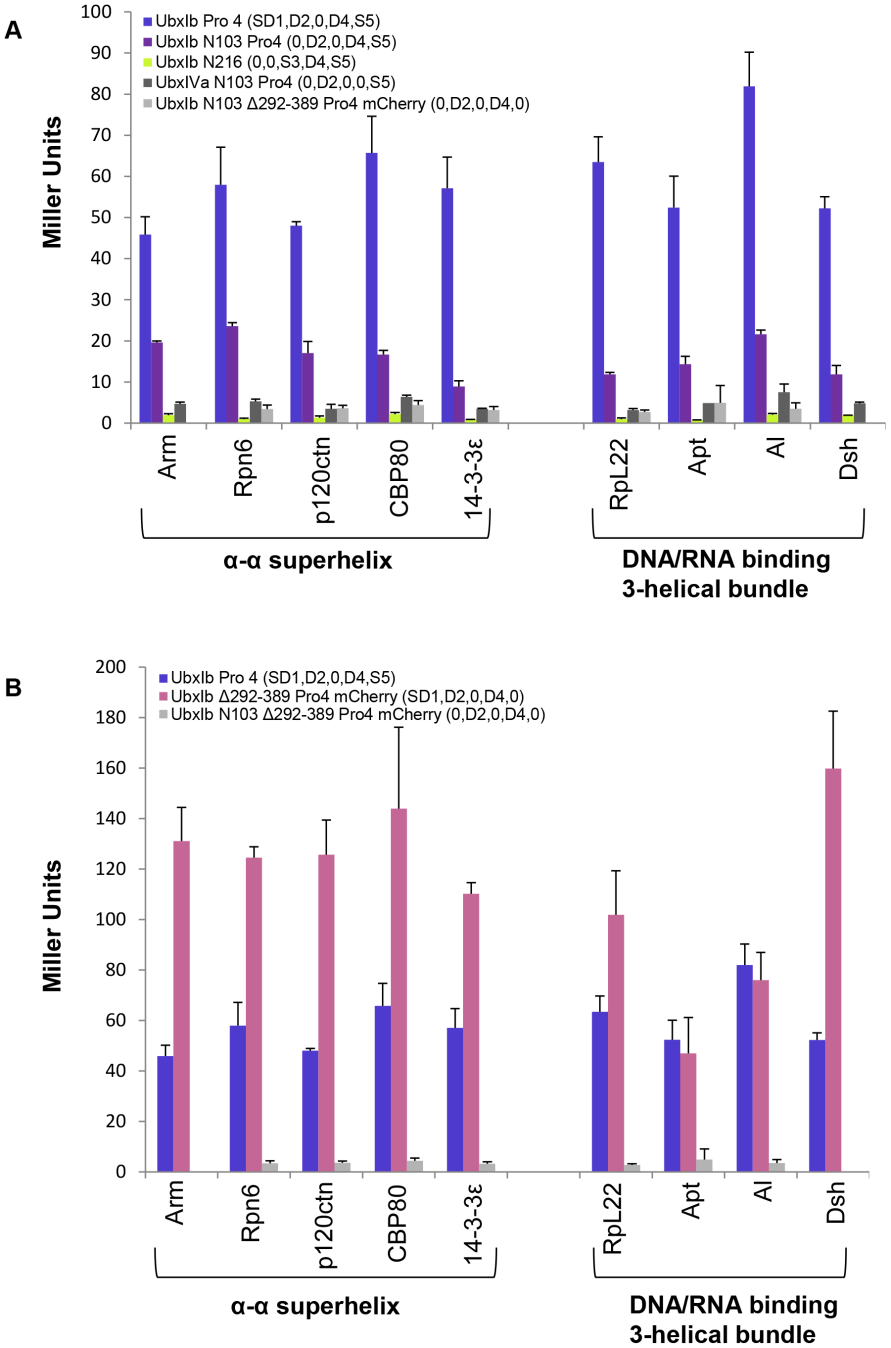
Defining minimal partner interaction domains. Analysis of yeast two-hybrid data using Ubx variants identifies two overlapping minimal partner interaction domains: UbxIb N103 Pro4 (0, D2,0, D4, S5) (Panel A) and UbxIb Δ292–389 Pro4 mCherry (SD1, D2,0, D4,0) (Panel B). Both minimal partner binding domains include the disordered Regions 2 and 4.

To try to identify a minimal region of Ubx required for protein interactions, we began with UbxIb N103 Pro4 (0, D2,0, D4, S5), a truncated variant which binds all partner proteins, and iteratively removed each remaining structured or disordered region (Figure 7A). UbxIb N216 (0,0, S3, D4, S5), which additionally removes the disordered Region 2, cannot bind any of the Ubx partners. Likewise, UbxIVa N103 Pro4 (0, D2,0,0, S5) which removes the disordered Region 4, cannot bind any of the Ubx partners. Finally, the structured C-terminus was removed in UbxIb N103 Δ292–389 Pro4 mCherry (0, D2,0, D4,0), which also cannot bind Ubx partners. Therefore Regions 2, 4, and 5 can be considered a minimal partner interaction region.

These data apparently conflict with data from the UbxIb Δ292–389 Pro4 mCherry (SD1, D2,0, D4,0) mutant, which also is able to bind all partners but lacks the S5 region in the minimal partner interaction region described above. Instead, this variant includes the SD1 region with mixed structure and disorder. Removal of the SD1 region to create UbxIb N103 Δ292–389 Pro4 mCherry (0, D2,0, D4,0) prevents binding to Ubx partners (Figure 7B). Therefore the UbxIb Δ292–389 Pro4 mCherry (SD1, D2,0, D4,0) variant constitutes a second minimal partner interaction region. The presence of two minimal partner interaction regions that are compatible with many Ubx-binding proteins may provide an opportunity for multiple partners to simultaneously bind Ubx. Inclusion of multiple binding sites has been observed for other disordered proteins [39]. The fact that both minimal partner binding regions are mainly composed of intrinsically disordered sequences highlights the important role that disorder plays in interactions mediated by Ubx.

### The intrinsically disordered regions in Ubx differentially contribute to partner binding

**Figure 8:**
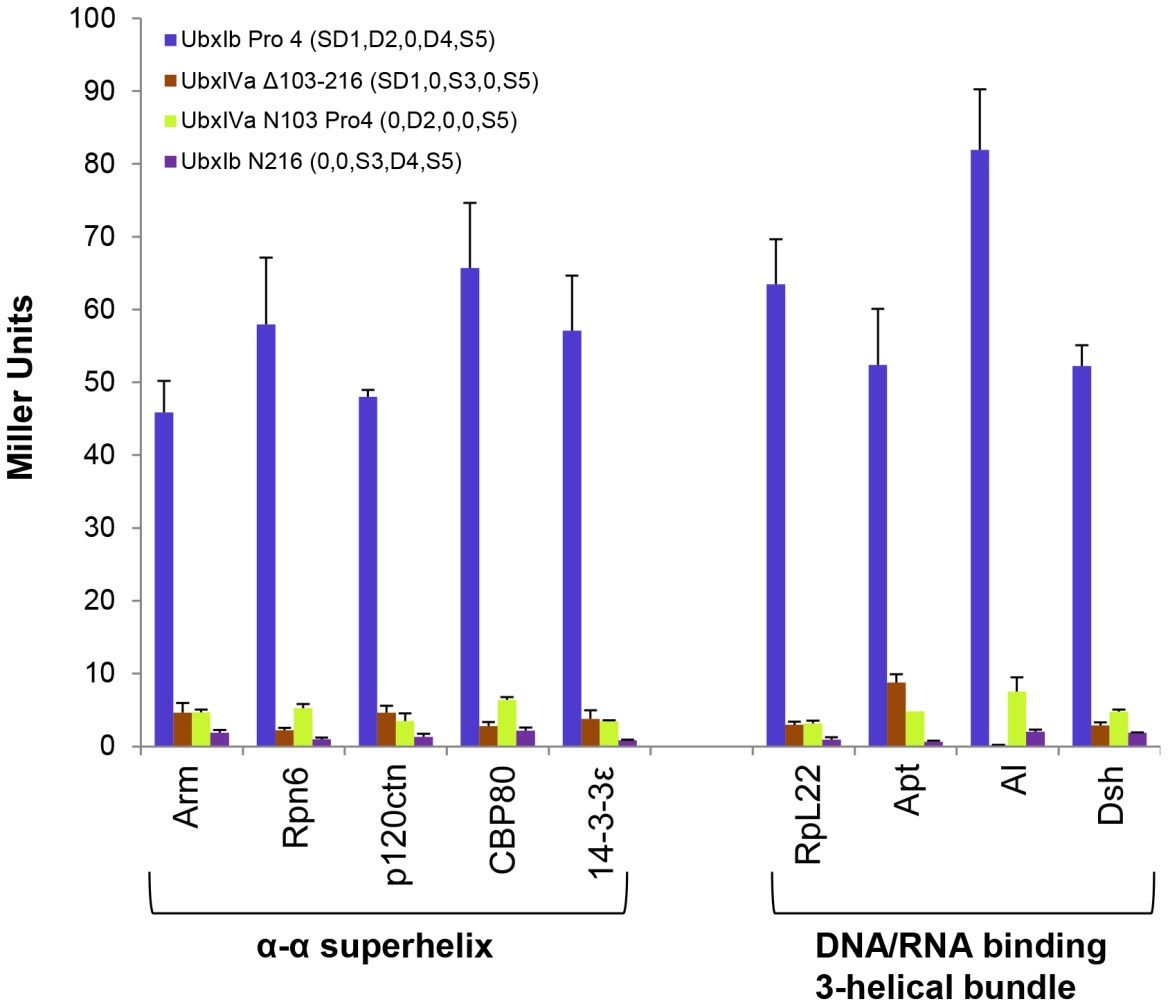
Ubx-interacting proteins cooperatively bind Regions 1, 2, and 3, all of which contain intrinsically disordered sequences. No single Ubx disordered region is sufficient to support partner binding, suggesting multiple disordered regions function as a cooperative unit.

Although the disordered regions are required for partner binding, different Ubx partner proteins may best interact with a subset of the Ubx disordered domains. If so, then identifying which intrinsically disordered region within Ubx is preferred by partner proteins could provide clues regarding the functional outcome of each partner interaction. For example, a partner that bound the Ubx transcription activation domain might alter the balance between transcription activation and repression by Ubx [42], [47]. Although the experiments described above suggest that the disordered regions are necessary for binding, they do not reveal which of the disordered regions are bound by partners. The most straightforward approach is to compare a Ubx variant with no disordered regions (UbxIVa N216) with a variant which includes just one of the disordered regions (Region 1, UbxIVa Δ103–216 (SD1,0, S3,0, S5); Region 2, UbxIVa N103 Pro4 (0, D2,0,0, S5); Region 4, UbxIb N216 (0,0, S3, D4, S5)). However, little to no partner binding was observed for all three of these variants, indicating more than one disordered region must be present for any partner to bind, consistent with the identification of the minimal binding regions described above (Figure 8).

To test the strength of different cooperative units, we compared variants missing each of the three disordered regions in turn (Region 1 deleted, UbxIb N103 Pro4 (0, D2, S3, D4, S5); Region 2 deleted, UbxIb Δ103–216 (SD1,0, S3, D4, S5); Region 4 deleted, UbxIVa Pro4 (SD1, D2, S3,0, S5). As already discussed, each of these mutants is still able to bind Ubx partner proteins. However, partner affinity is reduced to different extents (Figure 5). Binding by 14-3-3ε, RpL22, Apt, and Dsh was equally affected by removing Regions 1, 2, or 3. Since a large percentage (≥59%) of the signal was lost in each of these interactions, an interesting interpretation is that these proteins may simultaneously bind all three regions. For other partners, the magnitude of the reduction in protein interaction varies for the three regions. Whereas removing Regions 1 and 3 had a significant effect on binding all partners, for a subset of partners (*e.g*., p120ctn, Al, and CBP80), removing Region 2 had less impact. The ability of these three variants to bind partner proteins does not appear to correlate with the topology of the partner.

### Partners differentially interact with alternatively spliced isoforms of Ubx

Binding by all partners relies to some extent on contacts with Region 4, which contains sequences included in or excluded from Ubx by alternative mRNA splicing. Expression of Ubx splicing isoforms is regulated in a stage- and tissue-specific manner during *Drosophila* embryonic development [66]. Ubx spliceoforms are generated through differential inclusion of three different microexons in *ubx* mRNA, all of which code for protein sequences within Region 3: the b element, microexon I, and microexon II (Figure 1). Expression of these three splice variants elicits different phenotypes *in vivo* [67]–[69]. To determine the impact of alternative splicing on partner interactions, we compared the ability of UbxIb Pro4 (containing all three microexons), UbxIa Pro4 (containing the mI and mII microexons) and UbxIVa Pro4 (containing no microexons) to bind partner proteins.

**Figure 9:**
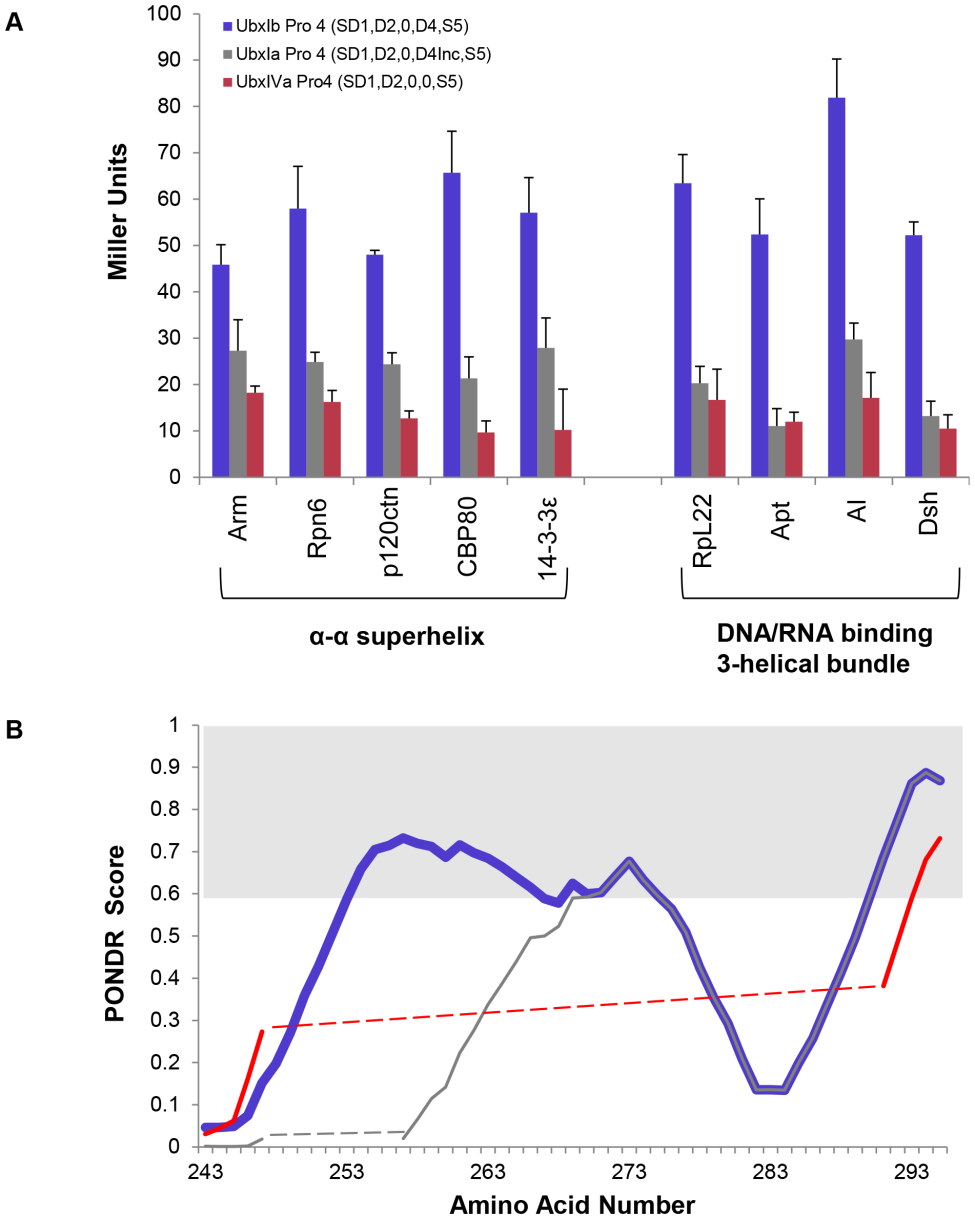
Ubx splicing isoforms are differentially able to bind partner proteins. Whereas all partners with an α-α superhelix fold bind UbxIa better than UbxIVa, among partners with a DNA/RNA binding 3-helical bundle fold only Al binds these two Ubx isoforms differently. “Inc” denotes Region 3, the microexon region, and is incomplete in the UbxIa splicing isoform. The disordered regions remaining in each variant are listed in parentheses after the protein name. (B) Colored lines represent intrinsic disorder prediction scores for the microexon region for different Ubx splicing isoforms, generated using the PONDR VL-XT algorithm [89], [90]. Blue line, UbxIb which has all 3 microexons; grey line, UbxIa which lacks the 9 amino acid b element; red line, UbxIVa, which lacks all 3 microexons. Dashed lines connect data across the microexon sequences removed in the shorter isoforms. The extent of predicted disorder (score>0.6, region shaded light grey) correlates with the ability to bind the tested partner proteins.

Removal of all three microexons in the UbxIVa Pro4 variant reduces the ability of Ubx to bind all partners relative to UbxIb Pro4 (Figure 9A). This reduction ranges from 85% (CBP80) to 60% of binding lost (Arm). For some partners (RpL22, Apt, and Dsh), removal of only the 9-amino acid b element altered binding to the same extent as removing all three microexons, indicating these interactions are critically dependent on the presence of the b element. We cannot discern from these experiments whether the b element contributes key chemical groups required for interaction or simply lengthens the intrinsically disordered region to generate a sufficiently large binding interface. Partner affinity has also been linked to the dynamics of the disordered region [3], [70]. Intriguingly, disorder prediction algorithms yield very different scores for different Ubx splicing isoforms (Figure 9B). These differences suggest that Ubx dynamics may influence Ubx-partner binding.

Partner topology generally correlates with partner affinity for different Ubx splicing isoforms. All proteins with an α-α superhelix fold bind UbxIa better than UbxIVa, whereas all but one protein (Al) with a DNA/RNA binding 3-helical bundle fold bind UbxIa and UbxIVa equally well (Figure 9). This correlation reflects similarities in binding by partners with the same fold. Interestingly, the Eukaryotic Linear Motif (ELM) prediction algorithm revealed a 14-3-3ε binding motif in the mII microexon sequence [71]–[74], which may explain why 14-3-3ε binds UbxIa Pro4, but not UbxIVa Pro4, which is missing this motif. In general, the proteins with a strong isoform effect (UbxIb>UbxIa>UbxIVa) were all negatively charged (14-3-3ε, Al, Arm, CBP80, p120ctn, and Rpn6), perhaps due to the position of the alternatively spliced microexons adjacent to the positively charged homeodomain. The previously characterized Ubx partner, Exd, also has a net negative charge and differentially binds Ubx isoforms [50]. Proteins that bind UbxIa and UbxIVa equally well can be either positively or negatively charged. Thus, although all partners bind disordered regions, the topology and charge of the partner protein correlate with their ability to bind different Ubx spliceoforms. Differences in the affinity of partners for Ubx spliceoforms create the potential for *ubx* mRNA splicing to regulate Ubx-partner interactions *in vivo*.

## Discussion

We have demonstrated that partner topology is a key aspect of protein interactions formed by the intrinsically disordered regions of the *Drosophila* Hox protein Ubx. Greater than 60% of Ubx-binding proteins have a fold in common with at least one other Ubx partner, and Ubx binds the selected fold within these proteins. Other laboratories have also identified disordered proteins that bind multiple partner proteins with similar structures [28], [75]. These partners were related proteins from the same protein family. In contrast, Ubx binds structurally similar, yet widely diverse proteins with very different chemical natures and molecular functions. Binding multiple partners with similar structures may reduce frustration in the Ubx-partner interface compared to interactions disordered proteins and an array of partner topologies [76].

### A model for the role of structure in Ubx-partner binding

Many proteins that interact with intrinsically disordered proteins or regions bind a MORF, a short motif within a disordered region of a protein that often folds upon partner binding. In the case of Ubx, three large disordered regions all simultaneously contribute to partner binding. The fact that the topology of the partner protein is important suggests that the disordered regions may need to be positioned in a specific manner in order to maximize interactions with the partner protein. This model fits with our data on the role of Regions 1 (partially structured) and 5 (structured) in partner binding. Neither structure-containing region is sufficient for partner binding, and partner binding can occur in the absence of either region. The inability of AkUbx, a natural Ubx orthologue which lacks most of the disordered regions, to bind partners demonstrates that the lack of binding is not an artifact induced by mis-positioning structured regions in Ubx mutants. However, either Region 1 or Region 5 must be present for the disordered regions in Ubx to bind partner proteins, suggesting either of these regions can correctly position the disordered domains for partner binding. This positioning may involve binding the disordered regions: the Ubx homeodomain, which is located in Region 5, has a DNA/RNA-binding 3-helix bundle fold, one of the two major folds selected by Ubx. The intrinsically disordered regions of Ubx directly interact with the homeodomain to alter its DNA binding affinity and specificity [6], [7] and with each other to enable cooperative DNA binding *in vivo* and materials formation *in vitro* [77], [78].

### Implications for Ubx function

The identification of partner-binding regions within Ubx, and the overlap of these regions with each other and with known functions or regulatory mechanisms, has important implications for regulating tissue-specific Ubx function *in vivo*. Whereas some partners bind all three regions to an equal extent (14-3-3ε, RpL22, Apt, and Dsh), other partners depend more heavily on Regions 1 and 3 for binding to Ubx (Arm, p120ctn, CBP80, and Al). Ubx partners reliant on the same regions of Ubx for binding may compete for binding to these regions.

For partners that bind equally well to all three intrinsically disordered regions, the long length of these regions may enable more than one partner to simultaneously bind Ubx. Indeed, other proteins with long disordered regions can act as a scaffold to simultaneously bind multiple partner proteins and create multi-functional complexes [29], [32], [79], [80]. In the context of transcription regulation, using Ubx as a scaffold for constructing a multi-protein transcription factor complex allows Ubx-mediated transcription regulation to respond to input from multiple protein systems [2]. The correct, tissue-specific regulatory complex would be stabilized by Ubx-DNA interaction, partner-DNA interactions, and partner-Ubx interactions.

All Ubx partners rely, to some extent, on Region 2 for binding. Since Region 2 includes critical sequences for transcription activation by Ubx [43], partner binding may modulate the ability of Ubx to activate transcription. Further, multiple phosphorylation sites exist within Regions 1 and 2 [81], suggesting that phosphorylation of this region *in vivo* has the potential to regulate Ubx activity by removing bound proteins, stabilizing protein interactions, and/or altering which proteins are bound to this region.

**Figure 10:**
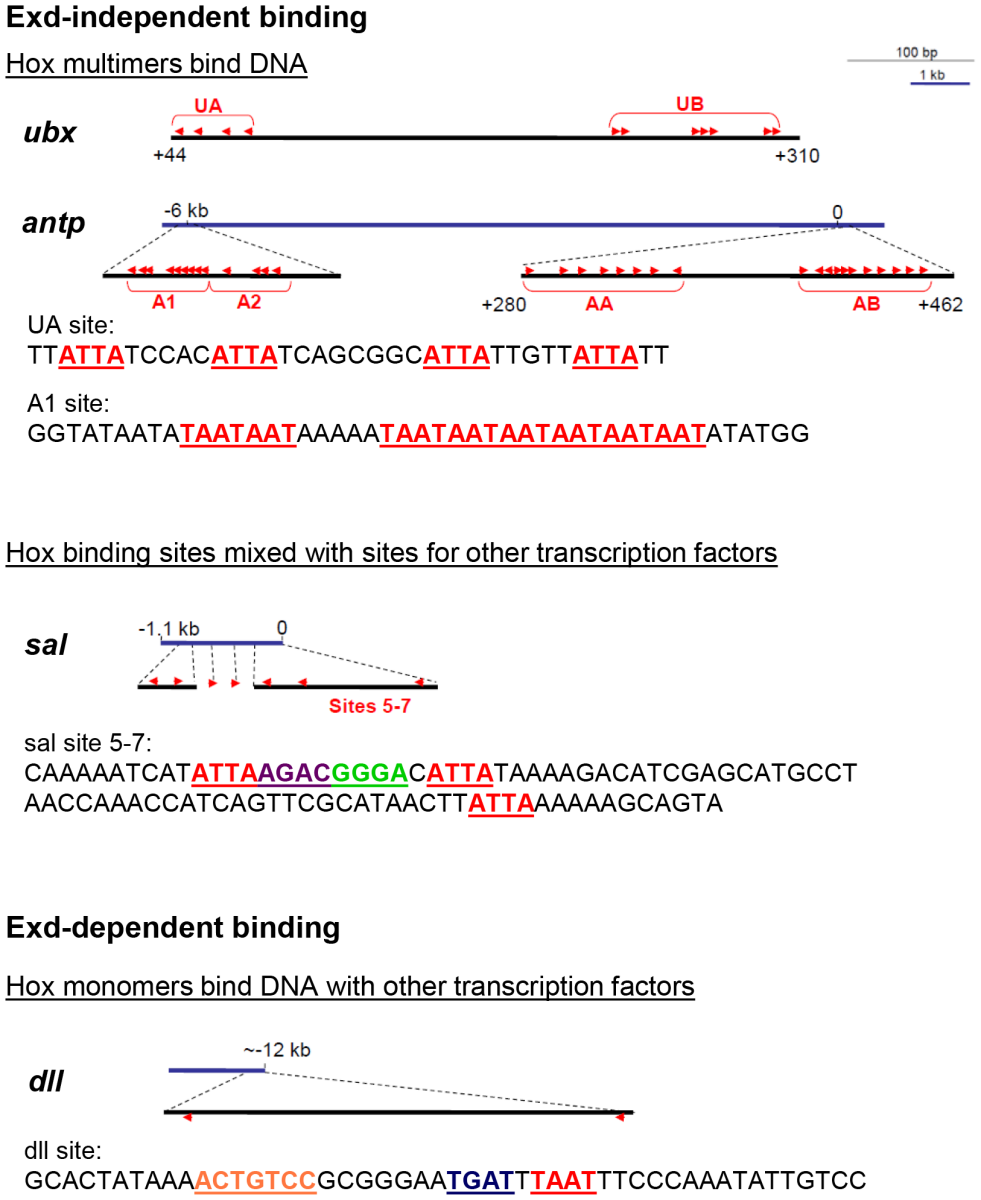
Ubx recognizes three categories of DNA binding sites. Ubx cooperatively binds multimers of Hox binding sites (TAAT/ATTA, red text), including enhancers for the *ubx* and *antp* genes [76]. Other transcription factors are not known to influence Ubx binding to these sites. In the second category, DNA binding sites for Ubx monomers are separated by DNA binding sites for other transcription factors (Medea, purple text, and Mad, green text) [91]. Regulation of the *sal* gene is coordinated by both Ubx and BMP signaling, which controls the activity of Medea and Mad. In the final category, Ubx binds DNA and regulates transcription in association with Exd (blue text) and Hth (orange text), general Hox co-factors [92], [93]. The positions of the DNA sequences are marked in bp relative to the start of transcription.

Alternative splicing alters the ability of Ubx to bind partners, a regulatory mechanism used to regulate the other protein interactions [82.83]. Alternative splicing, combined with protein partner availability may also impact how Ubx selects DNA binding sites. Ubx binds three different categories of DNA sequences, defined by the protein interactions in which Ubx engages: i) multiple, closely spaced Hox binding sites that permit cooperative Ubx binding, ii) single or multiple Hox binding sites interspersed with binding sites for other transcription factors, or iii) Hox-Exd heterodimer binding sites (Figure 10). The partner binding preferences of each Ubx isoform, combined with the presence or absence of partners in the tissues in which that isoform is expressed, could determine which subset of DNA sequences are regulated by Ubx in each tissue. For example, the presence of the b element enhances binding by the partners examined in this study, but reduces binding by Exd, the general Hox cofactor in *Drosophila* [50]. Thus, we would predict that UbxIa would preferentially bind Exd, and hence Hox-Exd heterodimer DNA binding sites, whereas UbxIb would preferentially interact with other transcription factors to regulate DNA sequences in which Ubx binding sites are interspersed with partner binding sites. Because these isoforms are expressed in the same tissues but not at the same levels [84], the relative concentrations of UbxIb and UbxIa may partition the available Ubx protein between genes regulated by Ubx-Exd heterodimers relative to genes regulated by Ubx in conjunction with other partner proteins. Likewise, the absence of partner proteins or the decreased affinity of partner proteins for a particular Ubx isoform, may direct Ubx to cooperatively bind DNA as homo-oligomers. Together, these mechanisms may contribute to isoform-specific differences in target gene recognition *in vivo* [67]–[69].

Finally, whereas the α-α superhelix partners bound specific disordered regions and Ubx isoforms better than others, the DNA/RNA binding 3-helical bundle fold partners tended to bind all three disordered regions equally well and bound UbxIa as well as UbxIVa. The reduced sequence specificity of DNA/RNA binding 3-helical proteins may reflect the fact that all of the disordered regions in Ubx evolved to interact with the Ubx homeodomain to regulate DNA binding [6], [7]. Since the homeodomain has a DNA/RNA binding 3-helical fold, the homeodomain-interacting disordered regions can also bind other proteins with this same fold. This hypothesis predicts that protein interactions may enhance DNA binding by removing the inhibitory disordered regions from the surface of the Ubx homeodomain. Conversely, DNA binding may facilitate Ubx-partner interactions by making the disordered regions more available for partner interactions. This scenario provides a mechanism, consistent with its cellular role, for Ubx to functionally integrate binding to a multiplicity of diverse protein partners and to DNA.

### Evolution of Hox function

The sequence of intrinsically disordered regions evolves more rapidly than for structured regions [85], [86], enabling incorporation of novel functions or binding modes. Indeed, the evolution of novel protein interaction motifs can change Hox function [63] or even dramatically transform a Hox protein into to a different class of transcription factor [88]. Based on our current knowledge, Ubx appears unlikely to interact with a subset of the proteins identified as binding partners for its natural *in vivo* function [49]. However, the ability of Ubx to bind functionally different proteins with similar structures may provide a mechanism to evolve novel Ubx functions. A new protein may be able to bind the disordered regions in Ubx based on its resemblance to an established Ubx partner, creating new modes of Ubx (or partner) regulation *in vivo*. As the Ubx sequence evolves, a specific motif for binding that partner may emerge, and with time eventually become an obligatory binding site. Examples of proteins at each of these stages may be found among the Ubx partner proteins. Most of the partners appear to recognize the disordered regions without any clear sequence or motif preferences, representing a relatively early stage in the evolution of partner binding. However, a 14-3-3ε interaction motif [71]–[74] occurs in the mII element of Ubx. Although the presence of this motif enhances 14-3-3ε binding, this protein still binds Ubx, albeit weakly, in the absence of this motif. Furthermore, the motif is located in a region of the Ubx protein for which inclusion depends on *ubx* mRNA splicing, allowing tissue-specific control of Ubx's affinity for 14-3-3ε. In our model, the enhancement, but not obligatory reliance, of partner binding by a recognition motif represents an intermediate stage of partner evolution. Finally, Exd/Pbx is an ancient Hox protein partner required for many basic Hox functions. Although the disordered regions in Ubx influence Exd binding, Exd interactions are primarily dependent on specific motifs in the Ubx sequence [87]. Exd binds different motifs in Ubx to elicit different functional outcomes *in vivo* [87]. Thus Ubx-Exd interactions represent a highly evolved partner interaction.

## Conclusions

We have demonstrated that the intrinsically disordered regions in Ubx select interacting partner proteins based, in part, on the topology of the protein partner. Furthermore, partner topology determines the affinity of binding to Ubx spliceoforms. The ability of multiple disordered regions in Ubx to bind numerous partners creates a variety of mechanisms for regulating partner binding, including competition or cooperation, preferences of alternatively spliced Ubx isoforms for specific protein – and thus DNA – interactions, and synergistic partner and DNA binding. The overlap of partner binding regions with functional or regulatory domains may provide an additional mechanism for partners to impact molecular functions such as transcription activation and DNA binding. Alteration of the Ubx disordered regions *via* phosphorylation and mRNA splicing provide opportunities for tissue-specific regulation of Ubx-partner interactions.

## Supporting Information

Figure S1 **Maps of a large-scale** ***Drosophila melanogaster*** **yeast two-hybrid data [51] parsed by fold, in which dots represents specific folds, and lines between dots depict interactions between the connected folds.** (A) All fold•fold interactions with a confidence score of at least 0.5 are shown. Intrafold interactions are depicted as loops which connect back to the originating node. (B) Mapping only fold•fold interactions with a confidence score of at least 0.5 and containing at least 3 protein•protein interactions significantly simplifies the depiction. The total number of protein interactions (for between 3 and 12 interactions) in one fold•fold connection is reflected in the weight of the lines. Connections with 12 or more interactions have the same line weight. Key folds discussed in the text are labeled on both maps.
(TIF)

Figure S2 **Probability distribution curves for** ***Drosophila*** **protein interactions from a large-scale yeast two-hybrid experiment parsed by fold.** Data were fit to a truncated scale-free model. The scatter observed at high k is often observed in scale-free systems [51], [94], [95]. The similarity of these graphs to each other and with the protein data [51] indicates that grouping data by structure do not alter network character. Graphs depicting the number of superfamilies, proportional to P(k), that have k interactions is shown as an inset. Deviations from a straight line in these graphs are indicative of biological restrictions on highly interactive proteins within a scale-free network.
(TIF)

Figure S3 **The distribution of the fold to interaction ratio (F/I) for (A) all single domain proteins and (B) all single domain proteins with more than one partner.** Proteins with a high ratio do not select protein partners by fold, whereas interactions with proteins with a low ratio have strong fold preferences. Ubx has an F/I ratio of 0.61, indicating a strong ability to select partners by fold.
(TIF)

Figure S4 **AkUbx, a Ubx orthologue with only one intrinsically disordered region, cannot bind** ***Drosophila*** **Ubx partners.** Sequence alignment between *Akanthokara kaputensis* Ubx (AkUbx) and *Drosophila melanogaster* Ubx showing the locations of disordered residues (red boxes) and the three disordered regions (blue labels).
(TIF)

Figure S5 **Ubx variants expression level does not correspond with partner interaction strength.** (A) Quantitative Western blotting result for Ubx variants protein expression in yeast (Strain:EGY48 transformed with p8op-LacZ reporter plasmid). (B) Weak correlation between yeast two-hybrid result and Ubx variants protein expression without outliers (R^2^ = 0.1403). Inset plot shows the influence of the two outliers (UbxIbN103 Pro4 Δ292–389 mCherry and UbxIb Δ292–389 Pro4 mCherry) on the correlation between yeast two-hybrid result and the Ubx variants protein expression.
(TIF)

Table S1 **Ubx partners with non-selected folds.** A fold with only one partner was classified as a non-selected fold. Folds for Ubx binding partners were classified according to SCOP.
(DOCX)
