# Supplementary figures and images for: The Intrinsically Disordered Regions of the Drosophila melanogaster Hox Protein Ultrabithorax Select Interacting Proteins Based on Partner Topology
Source: PLoS One. 2014 Oct 6;9(10):e108217. doi: 10.1371/journal.pone.0108217 (PMC4186791; doi:10.1371/journal.pone.0108217)

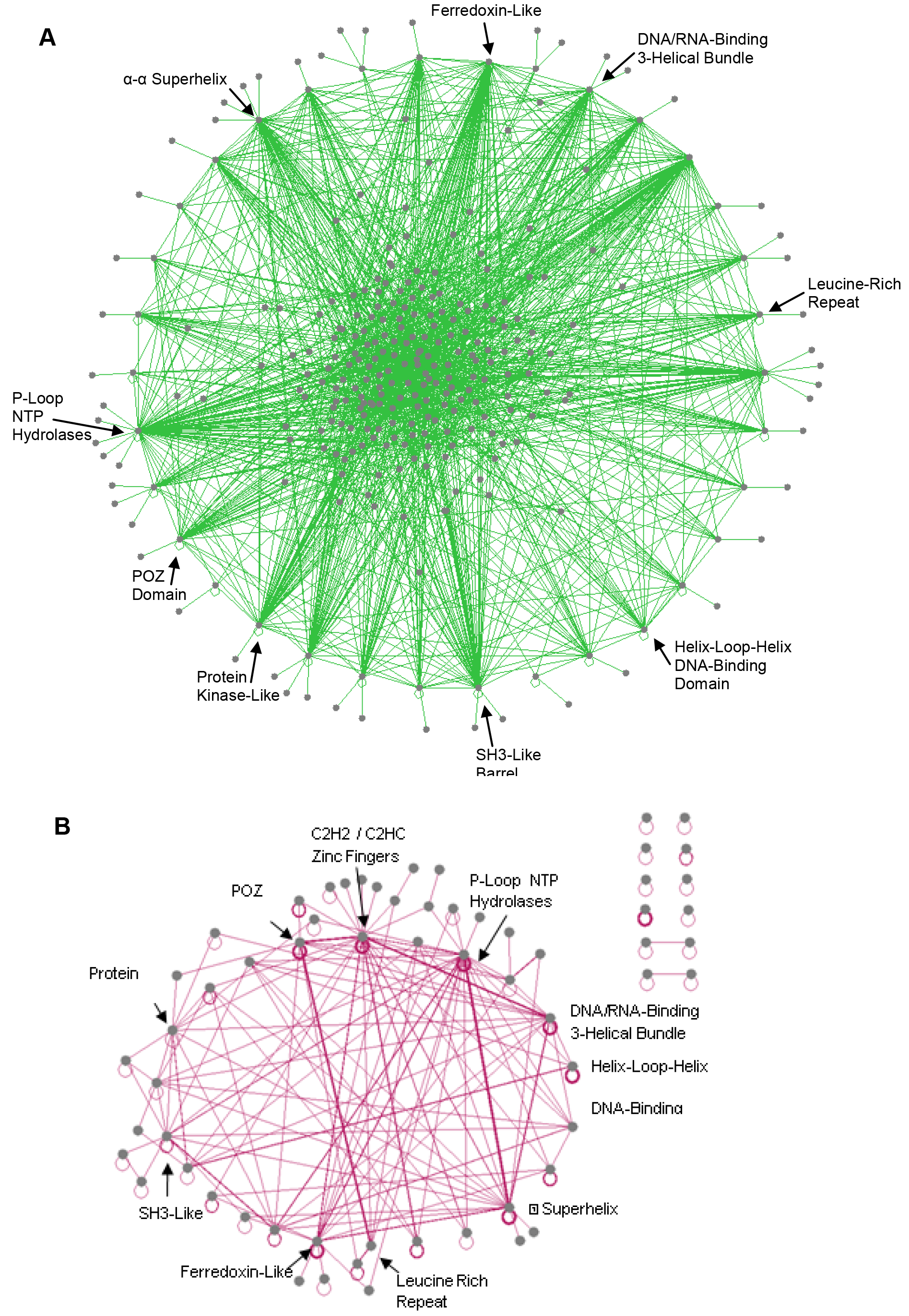

Supplement: Figure S1 — Maps of a large-scale Drosophila melanogaster yeast two-hybrid data [51] parsed by fold, in which dots represents specific folds, and lines between dots depict interactions between the connected folds. (A) All fold•fold interactions with a confidence score of at least 0.5 are shown. Intrafold interactions are depicted as loops which connect back to the originating node. (B) Mapping only fold•fold interactions with a confidence score of at least 0.5 and containing at least 3 protein•protein interactions significantly simplifies the depiction. The total number of protein interactions (for between 3 and 12 interactions) in one fold•fold connection is reflected in the weight of the lines. Connections with 12 or more interactions have the same line weight. Key folds discussed in the text are labeled on both maps. (TIF) [file pone.0108217.s001.tif]

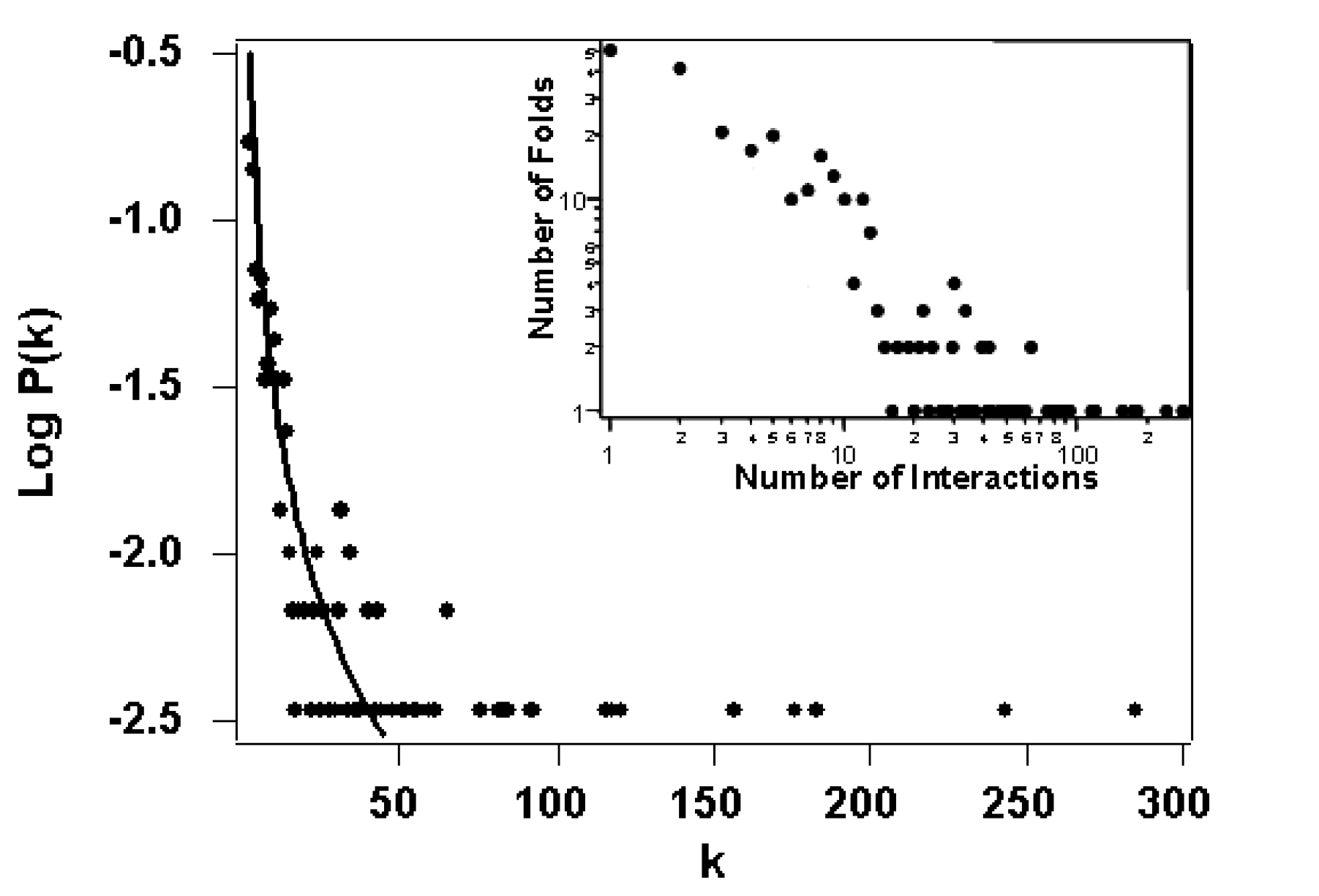

Supplement: Figure S2 — Probability distribution curves for Drosophila protein interactions from a large-scale yeast two-hybrid experiment parsed by fold. Data were fit to a truncated scale-free model. The scatter observed at high k is often observed in scale-free systems [51], [94], [95]. The similarity of these graphs to each other and with the protein data [51] indicates that grouping data by structure do not alter network character. Graphs depicting the number of superfamilies, proportional to P(k), that have k interactions is shown as an inset. Deviations from a straight line in these graphs are indicative of biological restrictions on highly interactive proteins within a scale-free network. (TIF) [file pone.0108217.s002.tif]

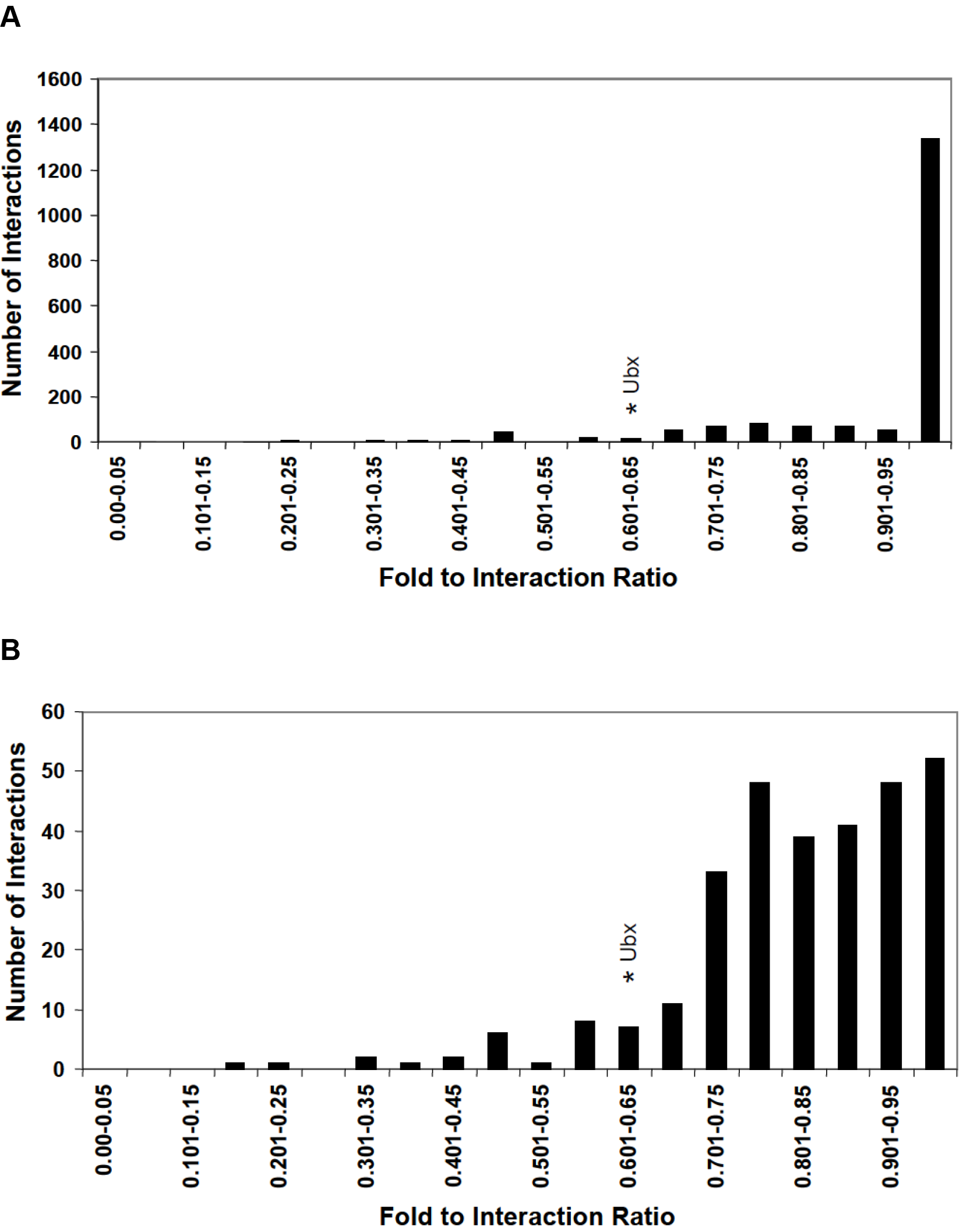

Supplement: Figure S3 — The distribution of the fold to interaction ratio (F/I) for (A) all single domain proteins and (B) all single domain proteins with more than one partner. Proteins with a high ratio do not select protein partners by fold, whereas interactions with proteins with a low ratio have strong fold preferences. Ubx has an F/I ratio of 0.61, indicating a strong ability to select partners by fold. (TIF) [file pone.0108217.s003.tif]

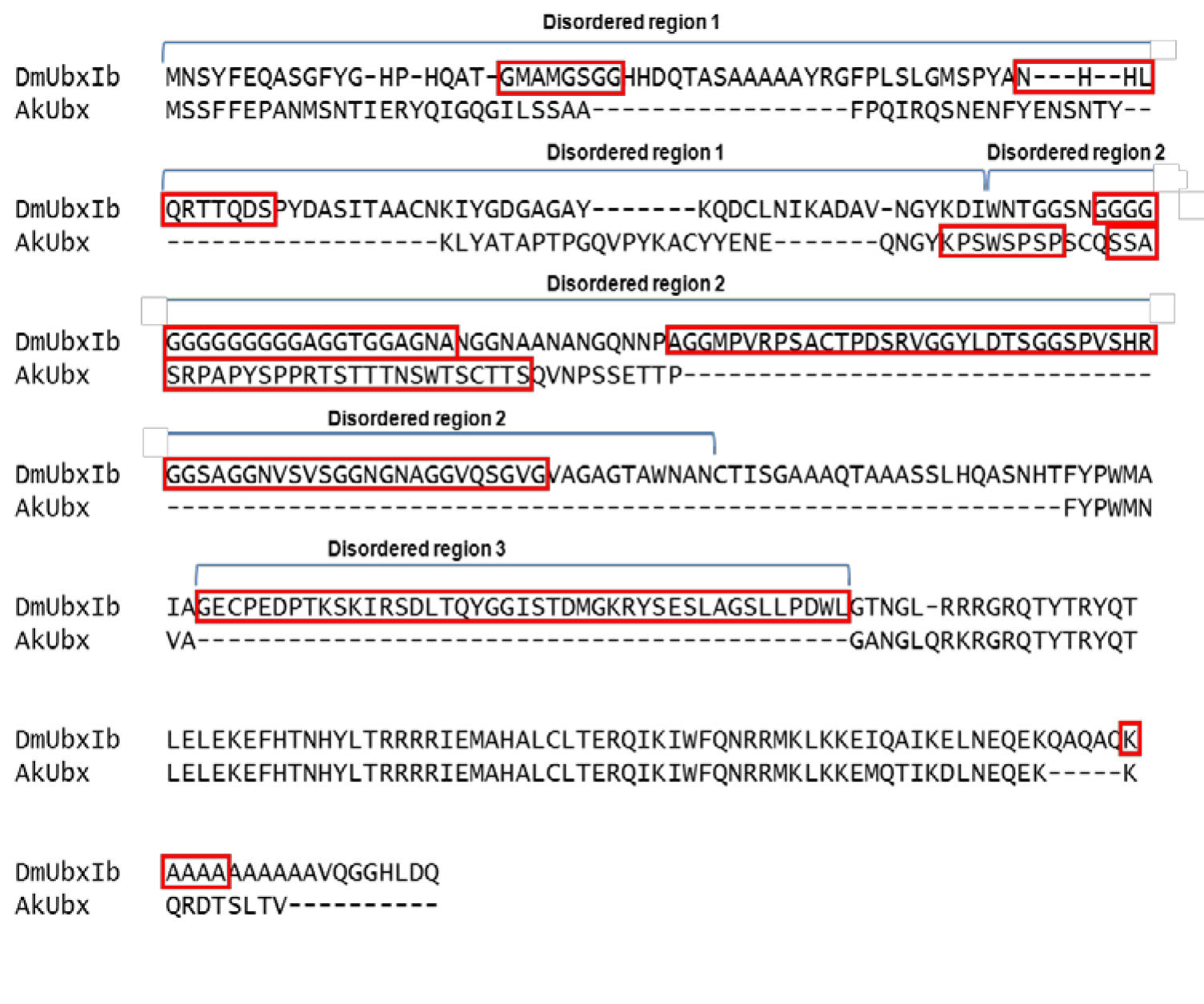

Supplement: Figure S4 — AkUbx, a Ubx orthologue with only one intrinsically disordered region, cannot bind Drosophila Ubx partners. Sequence alignment between Akanthokara kaputensis Ubx (AkUbx) and Drosophila melanogaster Ubx showing the locations of disordered residues (red boxes) and the three disordered regions (blue labels). (TIF) [file pone.0108217.s004.tif]

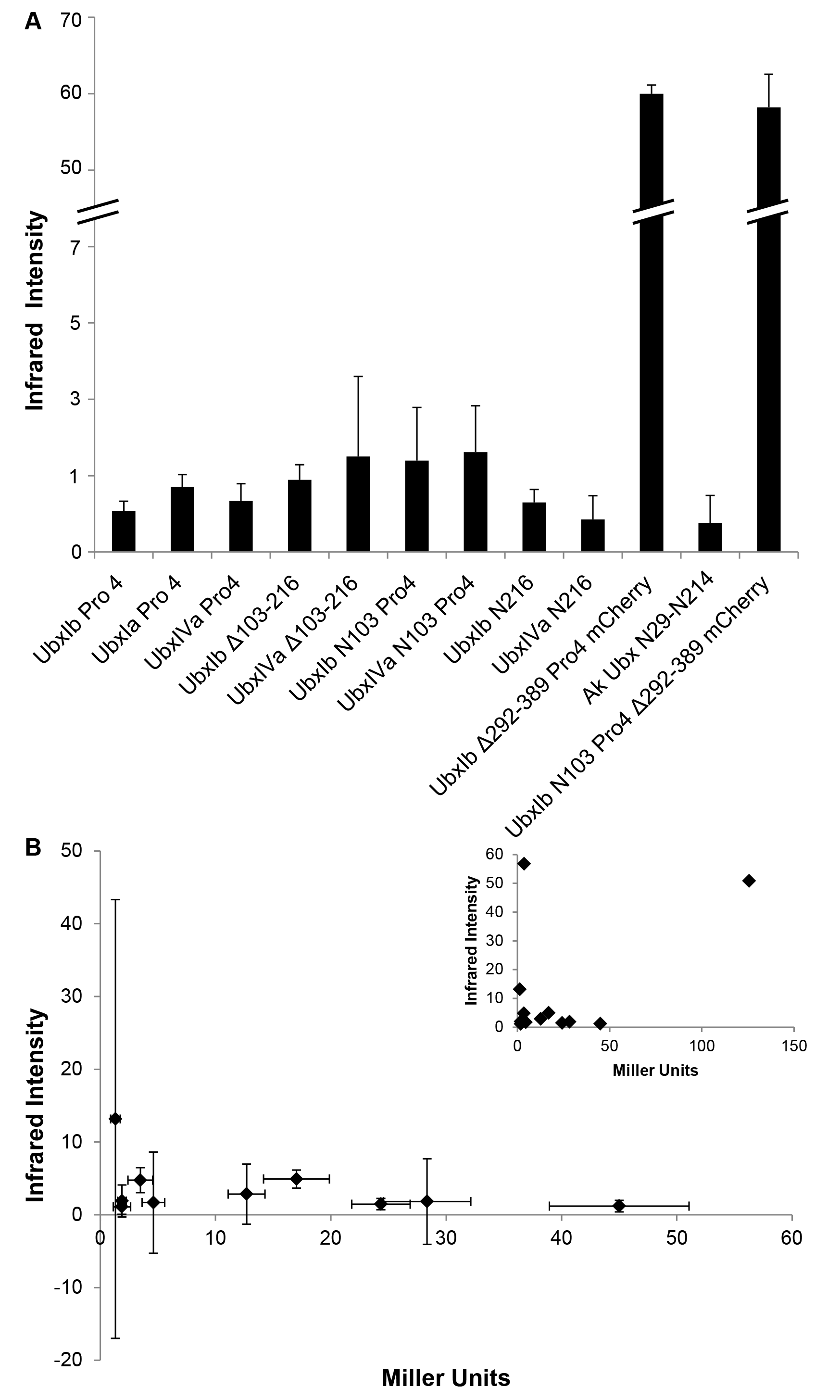

Supplement: Figure S5 — Ubx variants expression level does not correspond with partner interaction strength. (A) Quantitative Western blotting result for Ubx variants protein expression in yeast (Strain:EGY48 transformed with p8op-LacZ reporter plasmid). (B) Weak correlation between yeast two-hybrid result and Ubx variants protein expression without outliers (R2 = 0.1403). Inset plot shows the influence of the two outliers (UbxIbN103 Pro4 Δ292–389 mCherry and UbxIb Δ292–389 Pro4 mCherry) on the correlation between yeast two-hybrid result and the Ubx variants protein expression. (TIF) [file pone.0108217.s005.tif]
