# Supplementary material for: The Intrinsically Disordered Regions of the Drosophila melanogaster Hox Protein Ultrabithorax Select Interacting Proteins Based on Partner Topology
Source: PLoS One. 2014 Oct 6;9(10):e108217. doi: 10.1371/journal.pone.0108217 (PMC4186791; doi:10.1371/journal.pone.0108217)
Supplement: Table S1 — Ubx partners with non-selected folds. A fold with only one partner was classified as a non-selected fold. Folds for Ubx binding partners were classified according to SCOP. (DOCX) [file pone.0108217.s006.docx]

| **Fold** | **Partner** | **Fold** | **Partner** |
| --- | --- | --- | --- |
| **FYVE/PHD zinc finger** | Mi-2 | **Ribosomal protein S5 domain 2-like** | Ef2b |
| **HLH-like** | Hairy | **Ribonuclease H – like motif** | Hairy |
| **Cyclin-like** | CycK | **α/β-Hydrolases** | Neurotactin |
| **XPC-binding domain** | Rad23 | **Chromo domain-like** | Mi-2 |
| **S15/NS1 RNA Binding Domain** | RpS13 | **Smad/FHA domain** | SMOX |
| **β-Grasp** | Rad23 | **Reductase/isomerase/elongation factor common domain** | Ef2b |
| **Smad MH1 domain** | SMOX | **PDZ domain-like** | Dsh |
|  | | | |
